# CTI-related composite adiposity indices and future cardiovascular disease risk among middle-aged and older Chinese adults

**DOI:** 10.3389/fnut.2026.1849538

**Published:** 2026-06-04

**Authors:** Kangjie Tang, Xinyue Guo, Yingxin Lu, Wenqing Li, Haiwei Chen, Zeru Chen, Meng Wei

**Affiliations:** 1 Department of Clinical Medicine, The Second School of Clinical Medicine, Guangzhou Medical University, Guangzhou, China; 2 Department of Ophthalmological and Optometric Medicine, Beijing Tongren Hospital, Capital Medical University, Beijing, China; 3 Department of Medical Imaging, The Second School of Clinical Medicine, Guangzhou Medical University, Guangzhou, China; 4 Department of Obstetrics and Gynecology, Peking University Third Hospital, Beijing, China; 5 Cardiometabolic Medicine Center, Fuwai Hospital, National Center for Cardiovascular Diseases, Chinese Academy of Medical Sciences and Peking Union Medical College, Beijing, China; 6 Department of General Surgery, The First Affiliated Hospital of AnHui Medical University, Hefei, China

**Keywords:** adiposity indices, cardiovascular disease, C-reactive protein-triglyceride-glucose index, prospective cohort, risk prediction

## Abstract

**Background:**

Composite biomarkers integrating inflammation, glycolipid metabolism, and adiposity may improve cardiovascular risk stratification, but evidence remains limited for CTI-related composite adiposity indices. This study investigated their associations with incident cardiovascular disease (CVD) and predictive performance.

**Methods:**

This prospective cohort study included 5,290 adults aged ≥45 years without baseline CVD from the China Health and Retirement Longitudinal Study (CHARLS, 2011–2020). Four CTI-related composite indices were evaluated: CTI-body mass index (CTI-BMI), CTI-body roundness index (CTI-BRI), CTI-Waist-to-height ratio (CTI-WHtR), and CTI-weight-adjusted waist index (CTI-WWI). Associations with incident CVD were assessed using Kaplan–Meier analysis, Cox regression, restricted cubic splines, time-dependent receiver operating characteristic curves, concordance index (C-index) analyses, net reclassification improvement (NRI), integrated discrimination improvement (IDI), and sensitivity analyses.

**Results:**

During follow-up, 1,340 participants developed CVD, accounting for 25.33% of the study population. Kaplan–Meier analysis showed significant differences across tertiles for all four indices. After adjustment for demographic characteristics, lifestyle factors, clinical history, laboratory indicators, and hypertension status, all four indices remained positively associated with incident CVD. Compared with the lowest tertile, the highest tertile was associated with significantly higher CVD risk, with hazard ratios ranging from 1.37 to 1.58. Restricted cubic spline analyses showed statistically significant nonlinear associations for all indices, while exploratory two-piecewise Cox models did not establish definitive clinical thresholds. At 4-year follow-up, CTI-WHtR showed the highest area under the curve (AUC) value (0.564). Adding CTI-WHtR improved the 4-year AUC from 0.596 to 0.608, with an NRI of 0.053 and an IDI of 0.005. Sensitivity analyses yielded consistent results.

**Conclusion:**

CTI-based composite adiposity indices were independently associated with incident CVD, and CTI-WHtR showed the numerically highest standalone 4-year AUC. However, its incremental predictive improvement beyond the baseline model was modest, suggesting that CTI-WHtR may be interpreted as a supplementary risk marker rather than a standalone prediction tool.

## 1 Introduction

Cardiovascular disease (CVD) remains one of the primary causes of death and disability worldwide, placing a considerable economic and caregiving burden on healthcare systems (1, 2). CVD-related mortality is projected to continue increasing globally, and China in particular faces a heavy cardiovascular disease burden, with an estimated 330 million affected individuals and nearly 45% of all deaths attributed to CVD (3, 4). Although considerable progress has been made in decreasing CVD risk through interventions aimed at conventional risk factors like hypertension and dyslipidemia, the total disease burden continues to grow, especially among middle-aged and older individuals (5). Therefore, timely recognition of individuals with a high likelihood of adverse cardiovascular events, beyond traditional risk factor assessment, is of great clinical importance for guiding individualized primary prevention, improving healthcare resource allocation, and developing long-term health management strategies (6).

In contemporary clinical practice, commonly used tools for primary CVD risk assessment, such as the Framingham Risk Score and the Pooled Cohort Equations, are largely constructed on established risk factors, including age, sex, blood pressure, lipid parameters, and smoking status (7, 8). More recently, risk prediction models such as the Predicting Risk of Cardiovascular Disease EVENTs (PREVENT) equations, which build upon and are expected to update the Pooled Cohort Equations framework, Systematic COronary Risk Evaluation 2 (SCORE2)/SCORE2-Older Persons (SCORE2-OP), and the Prediction for Atherosclerotic Cardiovascular Disease Risk in China (China-PAR) model have further expanded CVD risk estimation across different populations and clinical settings (9–12). Despite their important clinical utility, these risk scores are generally based on predefined clinical predictors and may not fully capture the complex interplay among inflammation, glycolipid metabolism, insulin resistance, and adiposity-related pathophysiological processes (13). Evidence is accumulating that the initiation and progression of CVD are driven by multiple interacting mechanisms. Chronic low-grade inflammation is involved throughout the development of atherosclerosis, insulin resistance contributes to endothelial dysfunction, and obesity, particularly central obesity, stimulates adipose tissue to secrete pro-inflammatory cytokines, thereby aggravating metabolic inflammation and reinforcing a self-perpetuating cycle (14, 15). In addition to impairing glucose and lipid homeostasis, insulin resistance is often accompanied by increased circulating free fatty acids, thereby exacerbating endoplasmic reticulum stress and mitochondrial dysfunction while promoting the proliferation and migration of vascular smooth muscle cells (16, 17). Nevertheless, the application of composite biomarkers that can conveniently and effectively integrate these multidimensional pathophysiological insights remains limited within current clinical risk assessment frameworks.

Over recent years, composite biomarkers have received growing interest for their capacity to reflect several interconnected pathophysiological processes at the same time. Among these biomarkers, the C-reactive protein–triglyceride–glucose index (CTI) incorporates C-reactive protein (CRP) together with the major glycolipid metabolic parameters, triglycerides (TG) and fasting blood glucose (FBG), and reflects the degree of metabolic inflammatory status (18). Evidence suggests that CTI provides independent prognostic information in multiple pathological conditions, including non-alcoholic fatty liver disease, metabolic syndrome, and cardiovascular outcomes (19–21). However, CTI itself does not capture obesity, a critical cardiovascular risk factor. Adipose tissue in obese individuals, particularly visceral fat, secretes multiple pro-inflammatory factors that synergistically enhance the inflammatory-metabolic stress represented by CTI, establishing an “inflammation-metabolism-obesity” vicious cycle that accelerates the occurrence of cardiovascular events (22). Combining CTI with different dimensions of obesity measurements may more comprehensively capture the aforementioned synergistic pathogenic effects. Such composite indices may also provide a concise way to describe the coexistence of metabolic inflammation and adiposity burden under a unified marker, while allowing different adiposity dimensions to be compared within a common CTI framework. However, combining CTI and adiposity measures into a single composite index is not the only possible analytical strategy. Therefore, in this study, we further compared these composite indices with models in which CTI and the corresponding anthropometric indicators were entered as separate predictors. Body mass index (BMI) reflects general adiposity, whereas body roundness index (BRI), waist-to-height ratio (WHtR), and weight-adjusted waist index (WWI) capture different aspects of central adiposity or body shape. These anthropometric measures have been associated with CVD risk, partly due to their representation of visceral adiposity and the corresponding metabolic risk profile (23, 24). However, comparative studies on composite indices constructed from CTI and these different obesity metrics for CVD risk prediction remain limited. Furthermore, whether these composite indices exhibit non-linear associations or specific threshold effects, and whether they provide incremental predictive information beyond traditional risk models, remains a subject of systematic investigation based on large-scale prospective cohort studies.

To address this gap in the literature, this study investigated the relationships between four CTI-related composite indices—CTI-BMI, CTI-BRI, CTI-WHtR, and CTI-WWI—and the risk of incident CVD using data from a large nationwide prospective cohort of middle-aged and older adults enrolled in China Health and Retirement Longitudinal Study (CHARLS). The present study sought to assess the independent relationships between these composite indices and incident CVD, examine potential non-linear dose–response associations and exploratory turning points, and compare the predictive performance of the various indices for CVD risk, and assess the incremental predictive value of the best-performing index beyond a baseline covariate model. Through comprehensive statistical analyses, this study aims to evaluate the potential supplementary value of CTI-related composite adiposity indices for CVD risk stratification, while providing epidemiological evidence on the synergistic roles of inflammation, metabolism, and obesity in the pathogenesis of cardiovascular disease.

## 2 Methods

### 2.1 Study design and participants

Data for the present study were derived from CHARLS, a nationwide prospective longitudinal study involving Chinese adults aged 45 years or above, designed to obtain detailed information on sociodemographic features, economic status, and health-related factors. Using a multistage stratified probability sampling approach, the study completed its baseline investigation in 2011–2012. The study population comprised 17,705 middle-aged and older individuals sampled from 28 provinces, 150 districts or counties, and 450 villages nationwide, with follow-up investigations carried out in 2013, 2015, 2018, and 2020. Detailed information on the study design and sampling strategy of CHARLS has been reported in previous publications (25). Ethical approval for the CHARLS protocol was granted by the Institutional Review Board of Peking University (No. IRB00001052-11015), and written informed consent was obtained from all participants. In this study, data from the five survey waves spanning 2011 to 2020 were analyzed to evaluate the associations between CTI-related indicators and incident cardiovascular disease (CVD).

**Figure 1:**
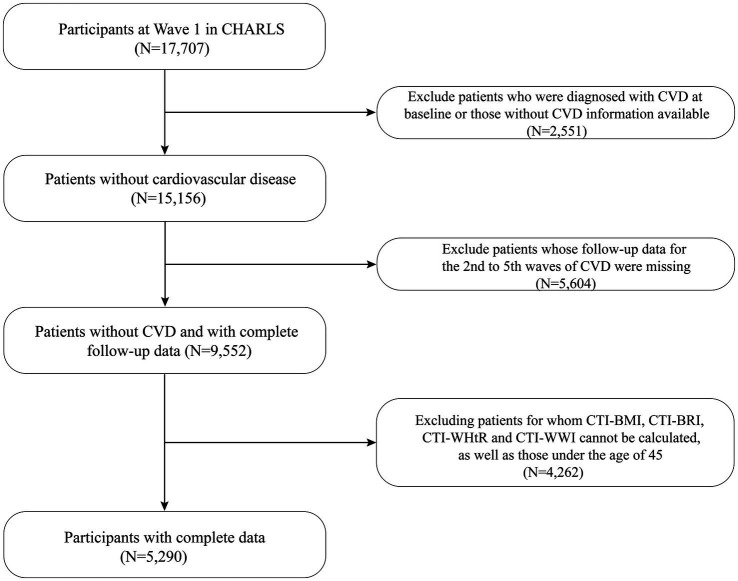
Flowchart of participant selection in the CHARLS cohort.

The following exclusion criteria were applied in this study: participants who had CVD at baseline or lacked baseline CVD information (*n* = 2,551); those with missing CVD follow-up data from the second to the fifth waves (*N* = 5,604); additional exclusions were made for individuals with missing information necessary to construct the CTI-related composite indices, specifically C-reactive protein, triglycerides, fasting blood glucose, height, waist circumference, and BMI, as well as for those aged under 45 years (*n* = 4,262). Following these exclusions, 5,290 participants remained in the final analytic sample, and the details of the selection procedure are illustrated in Figure 1. To evaluate the potential influence of participant exclusion on the study findings, baseline characteristics were compared between participants included in the analytic sample and those excluded because CTI-related composite indices could not be calculated or because they were younger than 45 years.

### 2.2 Calculation of the CTI and CTI-related composite indicators

CTI-BMI, CTI-BRI, CTI-WHtR, and CTI-WWI were constructed by multiplying the CTI by BMI, BRI, WHtR, and WWI, respectively. This multiplicative approach was adopted based on previous studies (26, 27) that constructed CTI-related adiposity indicators using the same method. Conceptually, these composite indicators integrate the inflammatory-metabolic status reflected by CTI with obesity-related information reflected by anthropometric indicators into a single marker. The formulas used to derive CTI, BRI, WHtR, and WWI are shown below (28–31):

$\mathrm{CTI}=0.412\times \mathrm{Ln}(\mathrm{CRP})+\frac{\ln(\mathrm{TG}\times \mathrm{FBG})}{2}$

$\mathrm{BRI}=364.2-365.5\times \sqrt{1-{\left(\frac{\frac{\mathrm{WC}}{2\pi }}{0.5\times \text{height}}\right)}^{2}}$

$\text{WHtR}=\frac{\mathrm{WC}}{\text{height}}$

$\mathrm{WWI}=\frac{\mathrm{WC}}{\sqrt{\text{weight}}}$

Where CTI denotes the C-reactive protein-triglyceride-glucose index, BMI body mass index, BRI body roundness index, WHtR waist-to-height ratio, WWI weight-adjusted waist index, CRP C-reactive protein, TG triglycerides, FBG fasting blood glucose, WC waist circumference, height body height, weight body weight, and ln the natural logarithm. CRP was expressed in mg/L, TG and FBG in mg/dL, WC and height in cm, and weight in kg.

### 2.3 Assessment of new-onset CVD events

The primary endpoint was incident CVD. Consistent with prior CHARLS-based studies (33), incident CVD was defined as the first report during follow-up of physician-diagnosed heart disease or stroke among participants without a CVD history at baseline. Baseline CVD was identified using the same questionnaire items, and participants who reported physician-diagnosed heart disease or stroke at baseline were excluded from the analytic cohort. This questionnaire-based ascertainment of CVD has been commonly used in previous epidemiological studies (34, 35) based on CHARLS, supporting the comparability of this outcome definition in CHARLS-based analyses.

Information on CVD events was collected at baseline and at follow-up waves 2 through 5 using standardized questionnaires. Specifically, participants were asked: “Have you ever been diagnosed by a doctor with myocardial infarction (MI), coronary heart disease (CHD), angina, congestive heart failure (CHF), or other heart problems?” and “Has a doctor ever confirmed that you had a stroke?” In this study, heart disease therefore included MI, CHD, angina, CHF, or other physician-diagnosed heart problems. Participants were considered to have incident CVD if they first reported any of the above heart diseases or stroke during the follow-up period.

For participants who developed CVD, follow-up time was calculated from baseline to the wave in which CVD was first reported. Participants who did not develop CVD were censored at their last available follow-up.

ICD-10 codes or medical-record-linked diagnostic codes were not available in the CHARLS public data used for this analysis. Therefore, CVD events were identified according to self-reported physician diagnoses rather than administrative diagnostic codes.

### 2.4 Assessments of covariates

Based on previous studies (18, 27, 29, 36) and clinical relevance, variables potentially associated with both CTI-related indicators and the occurrence of CVD were prespecified as covariates. Covariates were selected *a priori* according to previous CHARLS-based studies, clinical relevance, and data availability, rather than according to statistical significance in baseline comparisons. The fully adjusted model was used to control for potential confounding in the association analyses and was not intended to represent a validated CVD risk prediction score. All baseline covariate data were collected using the standardized CHARLS survey procedures, including sociodemographic characteristics, anthropometric measurements (33, 37), medical history, and laboratory tests. These factors were considered potential confounders and were incorporated into the multivariable models in the subsequent analyses.

### 2.5 Statistical analysis

Continuous variables were presented as mean ± standard deviation or median with interquartile range, according to their distribution, and were compared using analysis of variance or the Kruskal–Wallis test. Categorical variables were presented as counts and percentages and compared using the chi-square test. Baseline characteristics were compared between participants with and without incident CVD. To assess potential selection bias, baseline characteristics were also compared between participants included in and excluded from the analytic sample, and standardized mean differences were calculated.

Participants were categorized into tertiles according to each CTI-related composite index. Continuous CTI-related indices were standardized before regression analyses, and hazard ratios (HRs) were reported per 1-standard deviation (SD) increase to improve comparability across indices with different numerical scales. Kaplan–Meier curves and log-rank tests were used to compare cumulative hazard patterns across tertiles. Because incident CVD was identified at discrete CHARLS follow-up waves, these curves were interpreted as wave-based survival plots.

Associations between CTI-related composite indices and incident CVD were examined using Cox proportional hazards regression models. Model 1 was unadjusted. Model 2 was adjusted for age, sex, smoking status, and alcohol drinking status. Model 3 was further adjusted for marital status, educational attainment, place of residence, ethnicity, history of cancer, history of liver disease, total cholesterol, high-density lipoprotein cholesterol, uric acid, hemoglobin A1c, and serum creatinine. Model 4 was additionally adjusted for hypertension status, defined as self-reported physician-diagnosed hypertension and/or current antihypertensive medication use. The proportional hazards assumption was assessed using Schoenfeld residuals, and multicollinearity was examined using the generalized variance inflation factor.

Restricted cubic spline analyses were used to assess potential non-linear dose–response associations between CTI-related indices and incident CVD. Exploratory two-piecewise Cox regression analyses were further performed under the Model 4 framework to characterize the observed non-linear patterns. The identified points were interpreted as exploratory turning points rather than definitive clinical thresholds.

Time-dependent receiver operating characteristic curves and concordance index curves were used to compare the predictive performance of the CTI-related indices. The 4-year time point after baseline was selected as the primary time point for AUC comparison. Follow-up time was calculated from the baseline survey to the first report of incident CVD, death, or the last available follow-up visit through the 2020 survey, whichever occurred first. Participants without incident CVD were censored at their last available follow-up assessment. CHARLS Exit data were linked to identify deaths during follow-up; however, no death records were identified in the final complete-case analytic cohort. For the index with the strongest predictive performance, incremental predictive value beyond the baseline covariate model was assessed using changes in the area under the curve and concordance index, net reclassification improvement, and integrated discrimination improvement. The baseline covariate model included the covariates in Model 3.

To address whether the associations were mainly driven by CTI itself or by the anthropometric components, additional mutually adjusted Cox models were fitted by entering CTI and each anthropometric indicator simultaneously as separate predictors. The predictive performance of composite-score models was also compared with that of models including CTI and the corresponding anthropometric indicator as separate predictors, using 4-year area under the curve and Harrell concordance index.

Several sensitivity analyses were performed. First, the main Cox regression analyses were repeated with additional adjustment for lipid-lowering, antihypertensive, and antidiabetic medication use. Second, analyses were repeated after excluding participants with cancer or liver disease. Third, a parsimonious clinical risk-factor model including age, sex, smoking status, hypertension status, total cholesterol, high-density lipoprotein cholesterol, hemoglobin A1c, and serum creatinine was fitted to examine whether the findings were sensitive to extensive covariate adjustment. CHARLS Exit data were linked to identify deaths during follow-up; however, no death records were identified in the final complete-case analytic cohort, and therefore Fine–Gray competing-risk models were not fitted.

All analyses were performed using R version 4.2.2 and Free Statistics version 2.4. Two-sided *p* values < 0.05 were considered statistically significant.

## 3 Results

### 3.1 Participant baseline characteristics

After application of the eligibility criteria, 5,290 participants were included in the final analytic cohort. During follow-up, 1,340 participants developed incident CVD, corresponding to a cumulative incidence of 25.33%. Baseline characteristics according to incident CVD status are shown in Table 1.

**Table 1:** Baseline characteristics of participants grouped by with or without CVD.

Characteristic	Overall *N* = 5,290	Non-CVD *N* = 3,950	CVD *N* = 1,340	*p*-value
Age	58.06 ± 8.51	57.59 ± 8.50	59.45 ± 8.40	**< 0.001**
Sex				0.056
Female	2,909 (54.99)	2,142 (54.23)	767 (57.24)	
Male	2,381 (45.01)	1808 (45.77)	573 (42.76)	
Race				0.399
Others	326 (6.16)	237 (6.00)	89 (6.64)	
Han	4,964 (93.84)	3,713 (94.00)	1,251 (93.36)	
Marital status				0.639
Unmarried	500 (9.45)	369 (9.34)	131 (9.78)	
Married	4,790 (90.55)	3,581 (90.66)	1,209 (90.22)	
Education				0.326
Below primary school	3,702 (69.98)	2,750 (69.62)	952 (71.04)	
Junior school and above	1,588 (30.02)	1,200 (30.38)	388 (28.96)	
Residence place				0.254
Rural	3,625 (68.53)	2,690 (68.10)	935 (69.78)	
Urban	1,665 (31.47)	1,260 (31.90)	405 (30.22)	
Smoking status				< 0.001
Never	3,314 (62.65)	2,472 (62.58)	842 (62.84)	
Former	401 (7.58)	267 (6.76)	134 (10.00)	
Current	1,575 (29.77)	1,211 (30.66)	364 (27.16)	
Alcohol consumption				0.012
No	3,491 (65.99)	2,569 (65.04)	922 (68.81)	
Yes	1799 (34.01)	1,381 (34.96)	418 (31.19)	
Liver disease				< 0.001
No	5,148 (97.32)	3,863 (97.80)	1,285 (95.90)	
Yes	142 (2.68)	87 (2.20)	55 (4.10)	
Cancer				0.205
No	5,258 (99.40)	3,923 (99.32)	1,335 (99.63)	
Yes	32 (0.60)	27 (0.68)	5 (0.37)	
Taking prescription for cholesterol				< 0.001
No	5,077 (95.97)	3,841 (97.24)	1,236 (92.24)	
Yes	213 (4.03)	109 (2.76)	104 (7.76)	
Taking prescription for hypertension				< 0.001
No	4,481 (84.71)	3,451 (87.37)	1,030 (76.87)	
Yes	809 (15.29)	499 (12.63)	310 (23.13)	
Taking prescription for diabetes				< 0.001
No	5,148 (97.32)	3,869 (97.95)	1,279 (95.45)	
Yes	142 (2.68)	81 (2.05)	61 (4.55)	
Serological indicators				
HDL-C, mg/dL	194.89 ± 38.77	193.99 ± 38.93	197.56 ± 38.17	**0.004**
TC, mg/dL	51.77 ± 15.20	52.16 ± 15.18	50.62 ± 15.19	**0.001**
TG, mg/dL	101.78 (72.57, 147.79)	100.00 (71.68, 146.02)	107.97 (78.76, 155.76)	**< 0.001**
Scr, mg/dL	0.77 ± 0.18	0.77 ± 0.18	0.77 ± 0.18	0.583
Cystatin C, mg/dL	0.99 ± 0.23	0.99 ± 0.23	1.00 ± 0.23	0.262
UA, mg/dL	4.36 ± 1.20	4.37 ± 1.19	4.35 ± 1.21	0.684
HbA1c	5.26 ± 0.77	5.23 ± 0.70	5.36 ± 0.94	**< 0.001**
FBG, mg/dL	108.48 ± 32.42	107.35 ± 29.72	111.84 ± 39.15	**< 0.001**
BMI	23.52 ± 3.83	23.29 ± 3.71	24.18 ± 4.09	**< 0.001**
CTI	4.71 ± 0.57	4.68 ± 0.57	4.81 ± 0.57	**< 0.001**
BRI	4.13 ± 1.48	4.04 ± 1.44	4.39 ± 1.55	**< 0.001**
WWI	11.03 ± 1.29	10.99 ± 1.28	11.13 ± 1.32	**< 0.001**
CTI-BMI	111.46 ± 25.95	109.64 ± 25.24	116.83 ± 27.24	**< 0.001**
CTI-BRI	19.69 ± 8.14	19.13 ± 7.89	21.35 ± 8.62	**< 0.001**
CTI-WHtR	2.53 ± 0.54	2.49 ± 0.53	2.64 ± 0.56	**< 0.001**
CTI-WWI	52.05 ± 9.33	51.52 ± 9.20	53.60 ± 9.53	**< 0.001**

Continuous variables were presented as mean ± standard deviation (SD) or median [interquartile range (IQR)]; categorical variables were presented as weighted percentages and unweighted frequencies.

CVD, cardiovascular disease; BMI, body mass index; HDL-C, high-density lipoprotein cholesterol; TG, triglyceride; TC, total cholesterol; FBG, fasting blood glucose; HbA1c, hemoglobin A1c; WC, waist circumference; UA, uric acid; Scr, serum creatinine; CTI, C-reactive protein-triglycerides-glucose index; CTI-WHtR, C-reactive protein-triglycerides-glucose index - Waist-to-height ratio; CTI-BMI, C-reactive protein-triglycerides-glucose index - Body mass index; CTI-WWI, C-reactive protein-triglycerides-glucose index - Weight-adjusted waist index; CTI-BRI, C-reactive protein-triglycerides-glucose index - Body roundness index.

Compared with participants without incident CVD, those who developed CVD were older and had higher BMI values. They also differed in smoking status, alcohol drinking status, liver disease, antihypertensive medication use, lipid-lowering medication use, and antidiabetic medication use. Regarding laboratory and anthropometric indicators, participants with incident CVD had higher HbA1c, FBG, CTI, BRI, WWI, CTI-BMI, CTI-BRI, CTI-WHtR, and CTI-WWI values.

To assess potential selection bias, baseline characteristics were compared between participants included in and excluded from the analytic sample. As shown in Supplementary Table S1, excluded participants were slightly younger and had a higher proportion of men and participants with junior school education or above. The prevalence of hypertension and antihypertensive medication use was slightly lower among excluded participants. Although several variables differed statistically between the two groups, most standardized mean differences were small, suggesting that the magnitude of baseline imbalance was limited.

### 3.2 Cumulative CVD risk across tertiles of CTI-related composite indices

**Figure 2:**
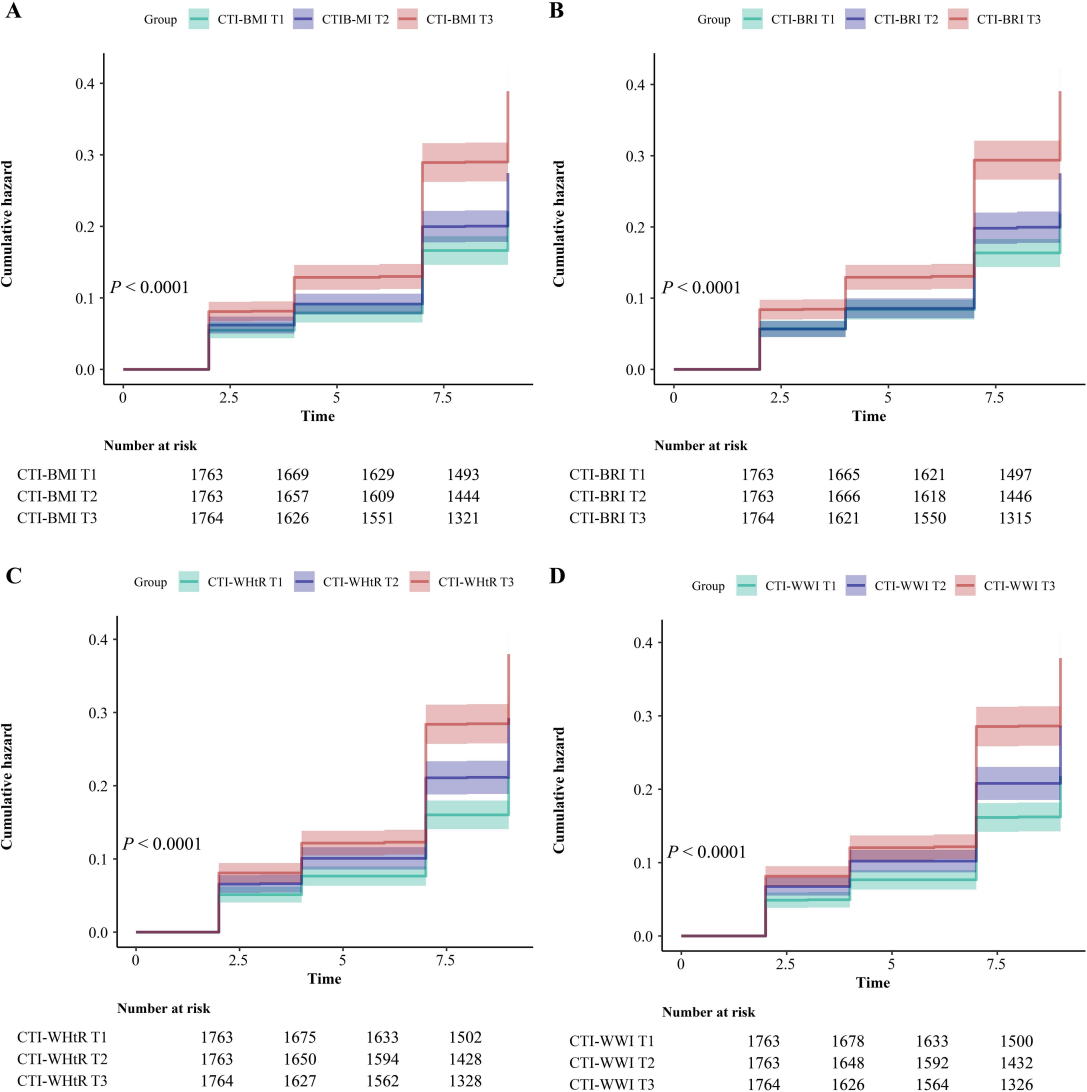
Kaplan–Meier curves for incident CVD according to tertiles of CTI-related composite indices. Kaplan–Meier curves showing cumulative hazard according to tertiles of CTI-related composite indices **(A–D)**. Group differences were assessed using the log-rank test. Since incident CVD was identified at discrete CHARLS follow-up waves, the step pattern reflects wave-based outcome ascertainment rather than continuous event recording. Numbers at risk are shown below each panel. Abbreviations: CTI, C-reactive protein-triglyceride-glucose index; BMI, body mass index; BRI, body roundness index; WHtR, waist-to-height ratio; WWI, weight-adjusted waist index; CVD, cardiovascular disease.

The Kaplan–Meier curves showed clear separation across tertiles of the CTI-related composite indices (Figure 2). Because incident CVD was assessed at discrete CHARLS follow-up waves, the curves displayed step patterns corresponding to the timing of follow-up assessments and should be interpreted as wave-based survival plots. Participants in the highest tertile consistently showed the greatest cumulative hazard, whereas those in the lowest tertile showed the lowest cumulative hazard.

Specifically, similar patterns were observed for CTI-BMI (Figure 2A), CTI-BRI (Figure 2B), CTI-WHtR (Figure 2C), and CTI-WWI (Figure 2D). Participants in the highest tertile (T3) generally showed the highest cumulative risk of CVD, whereas those in the lowest tertile (T1) showed the lowest risk. Log-rank tests indicated significant differences across tertiles for all four indices (all *p* < 0.0001).

### 3.3 Multivariable associations between CTI-related composite indices and incident CVD

The Cox regression results are presented in Table 2. In Model 4, which was additionally adjusted for hypertension status, all four CTI-related composite indices remained positively associated with incident CVD when modeled as standardized continuous variables. Each 1-SD increase in CTI-BMI, CTI-BRI, CTI-WHtR, and CTI-WWI was associated with increased CVD risk, with HRs of 1.19 (95% CI: 1.12–1.27), 1.15 (95% CI: 1.08–1.22), 1.15 (95% CI, 1.08–1.23), and 1.08 (95% CI, 1.01–1.15), respectively.

**Table 2:** Associations between CTI-related indicators and the risk of CVD.

Variables	Model 1		Model 2		Model 3		Model 4	
	HR (95% CI)	*P* value	HR (95% CI)	*P* value	HR (95% CI)	*P* value	HR (95% CI)	*P* value
CTI-BMI, per 1-SD increase	1.24 (1.18 ~ 1.29)	<0.001	1.24 (1.19 ~ 1.30)	<0.001	1.25 (1.18 ~ 1.33)	<0.001	1.19 (1.12 ~ 1.27)	<0.001
CTI-BMI terciles								
T1	1.0 [Ref]		1.0 [Ref]		1.0 [Ref]		1.0 [Ref]	
T2	1.24 (1.07 ~ 1.43)	0.003	1.26 (1.09 ~ 1.45)	0.002	1.25 (1.08 ~ 1.45)	0.003	1.21 (1.04 ~ 1.4)	0.012
T3	1.75 (1.53 ~ 2.00)	<0.001	1.80 (1.57 ~ 2.06)	<0.001	1.77 (1.51 ~ 2.07)	<0.001	1.58 (1.34 ~ 1.85)	<0.001
*P-*trend	1.33 (1.25 ~ 1.42)	<0.001	1.35 (1.26 ~ 1.44)	<0.001	1.34 (1.23 ~ 1.45)	<0.001	1.26 (1.16 ~ 1.36)	<0.001
CTI-BRI, per 1-SD increase	1.25 (1.19 ~ 1.32)	<0.001	1.23 (1.17 ~ 1.30)	<0.001	1.20 (1.13 ~ 1.28)	<0.001	1.15 (1.08 ~ 1.22)	<0.001
CTI-BRI terciles								
T1	1.0 [Ref]		1.0 [Ref]		1.0 [Ref]		1.0 [Ref]	
T2	1.25 (1.08 ~ 1.44)	0.002	1.24 (1.07 ~ 1.43)	0.004	1.21 (1.05 ~ 1.40)	0.010	1.15 (1.00 ~ 1.34)	0.055
T3	1.78 (1.56 ~ 2.03)	<0.001	1.72 (1.50 ~ 1.98)	<0.001	1.64 (1.41 ~ 1.92)	<0.001	1.48 (1.26 ~ 1.73)	<0.001
*P-*trend	1.34 (1.25 ~ 1.43)	<0.001	1.32 (1.23 ~ 1.41)	<0.001	1.29 (1.19 ~ 1.39)	<0.001	1.22 (1.13 ~ 1.32)	<0.001
CTI-WHtR, per 1-SD increase	1.26 (1.20 ~ 1.33)	<0.001	1.24 (1.17 ~ 1.30)	<0.001	1.21 (1.14 ~ 1.30)	<0.001	1.15 (1.08 ~ 1.23)	<0.001
CTI-WHtR terciles								
T1	1.0 [Ref]		1.0 [Ref]		1.0 [Ref]		1.0 [Ref]	
T2	1.38 (1.20 ~ 1.59)	<0.001	1.34 (1.16 ~ 1.54)	<0.001	1.32 (1.14 ~ 1.53)	<0.001	1.28 (1.10 ~ 1.48)	0.001
T3	1.79 (1.56 ~ 2.04)	<0.001	1.70 (1.48 ~ 1.96)	<0.001	1.62 (1.38 ~ 1.90)	<0.001	1.47 (1.25 ~ 1.73)	<0.001
*P-*trend	1.33 (1.25 ~ 1.42)	<0.001	1.30 (1.22 ~ 1.39)	<0.001	1.27 (1.17 ~ 1.38)	<0.001	1.21 (1.11 ~ 1.31)	<0.001
CTI-WWI, per 1-SD increase	1.22 (1.15 ~ 1.29)	<0.001	1.17 (1.11 ~ 1.24)	<0.001	1.12 (1.04 ~ 1.19)	0.001	1.08 (1.01 ~ 1.15)	0.017
CTI-WWI terciles								
T1	1.0 [Ref]		1.0 [Ref]		1.0 [Ref]		1.0 [Ref]	
T2	1.31 (1.14 ~ 1.51)	<0.001	1.25 (1.09 ~ 1.45)	0.002	1.23 (1.06 ~ 1.42)	0.006	1.20 (1.04 ~ 1.39)	0.013
T3	1.73 (1.52 ~ 1.98)	<0.001	1.59 (1.39 ~ 1.83)	<0.001	1.47 (1.26 ~ 1.72)	<0.001	1.37 (1.17 ~ 1.60)	<0.001
*P-*trend	1.32 (1.23 ~ 1.41)	<0.001	1.26 (1.18 ~ 1.35)	<0.001	1.21 (1.12 ~ 1.31)	<0.001	1.17 (1.08 ~ 1.26)	<0.001

Model 1: no covariates were adjusted.

Model 2: Sex, Age, Smoking, Drinking were adjusted.

Model 3: Sex, Age, Smoking, Drinking, Marital status, Education, Residence place, Race, Cancer, Liver disease, TC, HDL, UA, HbA1c, Scr.

Model 4: Sex, Age, Smoking, Drinking, Marital status, Education, Residence place, Race, Cancer, Liver disease, TC, HDL, UA, HbA1c, Scr, Hypertension status.

Abbreviations: CVD, cardiovascular disease; BMI, body mass index; HDL-C, high-density lipoprotein cholesterol; TG, triglyceride; TC, total cholesterol; FBG, fasting blood glucose; HbA1c, hemoglobin A1c; WC, waist circumference; UA, uric acid; Scr, serum creatinine; CTI, C-reactive protein-triglycerides-glucose index; CTI-WHtR, C-reactive protein-triglycerides-glucose index - Waist-to-height ratio; CTI-BMI, C-reactive protein-triglycerides-glucose index - Body mass index; CTI-WWI, C-reactive protein-triglycerides-glucose index - weight-adjusted waist index; CTI-BRI, C-reactive protein-triglycerides-glucose index - Body roundness index. HR, hazard ratio; CI, confidence interval; Ref, reference.

Similar associations were observed in tertile-based analyses. Compared with participants in the lowest tertile, those in the highest tertile had a significantly higher risk of incident CVD for CTI-BMI (HR = 1.58, 95% CI: 1.34–1.85), CTI-BRI (HR = 1.48, 95% CI: 1.26–1.73), CTI-WHtR (HR = 1.47, 95% CI: 1.25–1.73), and CTI-WWI (HR = 1.37, 95% CI: 1.17–1.60). Significant positive trends were observed across tertiles for all four indices (all *P* for trend < 0.001).

Model diagnostics supported the robustness of the Cox models. No evident multicollinearity among covariates was observed (Supplementary Table S2), and the proportional hazards assumption was not violated overall (GLOBAL *p* = 0.510, Supplementary Table S3).

### 3.4 Component-specific analyses of CTI and anthropometric indicators

Mutually adjusted Cox models were fitted by entering CTI and each anthropometric indicator simultaneously as separate predictors (Supplementary Table S4).

In the CTI + BMI model, both CTI and BMI were significantly associated with incident CVD after mutual adjustment. The HR was 1.08 for CTI (95% CI: 1.01–1.15, *p* = 0.022) and 1.16 for BMI (95% CI: 1.10–1.22, *p* < 0.001). In the CTI + BRI model, the mutually adjusted HRs were 1.09 for CTI (95% CI: 1.02–1.16, *p* = 0.011) and 1.12 for BRI (95% CI: 1.05–1.19, *p* < 0.001). In the CTI + WHtR model, the HRs were 1.09 for CTI (95% CI: 1.02–1.16, *p* = 0.009) and 1.11 for WHtR (95% CI: 1.04–1.18, *p* = 0.001). In the CTI + WWI model, CTI remained significant (HR = 1.10, 95% CI: 1.03–1.17, *p* = 0.003), whereas WWI was not statistically significant after adjustment for CTI (HR = 1.02, 95% CI: 0.96–1.08, *p* = 0.489).

### 3.5 Nonlinear dose–response associations and exploratory turning points

**Figure 3:**
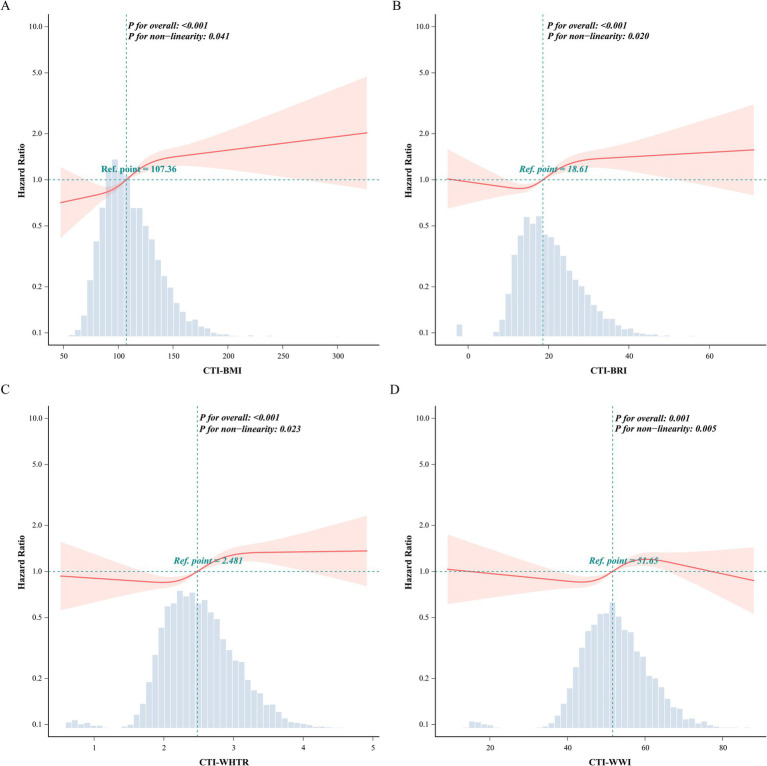
Restricted cubic spline analyses of the associations between CTI-related composite indices and incident cardiovascular disease. Restricted cubic spline analyses showing the dose–response relationships between CTI-related composite indices and incident CVD risk after further adjustment for hypertension status. **(A)** CTI-BMI; **(B)** CTI-BRI; **(C)** CTI-WHtR; **(D)** CTI-WWI. Red solid lines indicate adjusted hazard ratios, and shaded areas represent 95% confidence intervals. Histograms at the bottom of each panel show the distribution of each index. The vertical dashed lines indicate the identified points used in the exploratory two-piecewise Cox regression analyses and were also used as reference points for HR estimation in the RCS plots. These identified points should be interpreted as exploratory rather than definitive clinical thresholds. Abbreviations: HR, hazard ratio; CI, confidence interval; CTI, C-reactive protein-triglyceride-glucose index; BMI, body mass index; BRI, body roundness index; WHtR, waist-to-height ratio; WWI, weight-adjusted waist index; CVD, cardiovascular disease.

RCS analyses were used to evaluate the dose–response associations between CTI-related composite indices and incident CVD after further adjustment for hypertension status (Figure 3). The overall associations were statistically significant for all four indices. The p values for overall association were <0.001 for CTI-BMI, <0.001 for CTI-BRI, <0.001 for CTI-WHtR, and 0.001 for CTI-WWI. The p values for nonlinearity were 0.041 for CTI-BMI, 0.020 for CTI-BRI, 0.023 for CTI-WHtR, and 0.005 for CTI-WWI.

The identified reference points shown in Figure 3 were 107.36 for CTI-BMI, 18.61 for CTI-BRI, 2.481 for CTI-WHtR, and 51.65 for CTI-WWI. Exploratory two-piecewise Cox regression analyses were further performed using these reference points, and the results are shown in Supplementary Table S5. Positive associations above the reference points were observed for CTI-BRI and CTI-WHtR, whereas the log-likelihood ratio tests did not show statistically significant improvement of the two-piecewise models over the single-line models.

### 3.6 Standalone predictive performance of CTI-related indices

**Figure 4:**
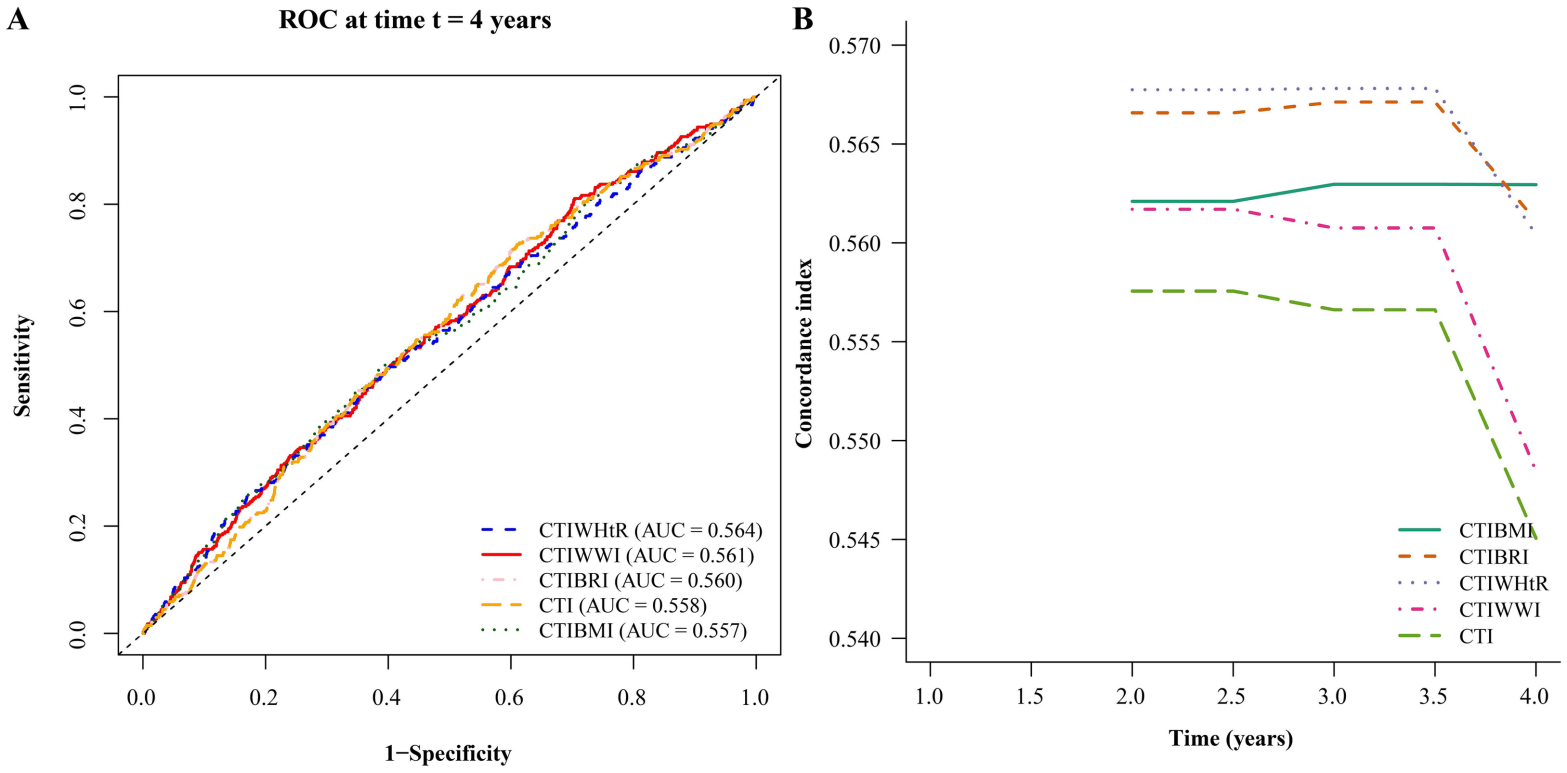
Predictive performance of CTI-related composite indices for incident cardiovascular disease. Comparison of the predictive performance of CTI-related composite indices for incident CVD **(A)** Time-dependent receiver operating characteristic (ROC) curves at 4 years for CTI-BMI, CTI-BRI, CTI-WHtR, CTI-WWI, and CTI alone. The area under the curve (AUC) for each index is displayed in the corresponding panel **(B)** Time-dependent concordance index (C-index) curves over follow-up. Abbreviations: ROC, receiver operating characteristic; AUC, area under the curve; C-index, concordance index; CTI, C-reactive protein-triglyceride-glucose index; BMI, body mass index; BRI, body roundness index; WHtR, waist-to-height ratio; WWI, weight-adjusted waist index; CVD, cardiovascular disease.

The standalone predictive performance of CTI-related indices for incident CVD was evaluated using time-dependent receiver operating characteristic curves and C-index analyses (Figure 4). At the 4-year time point, the AUC values were 0.557 for CTI-BMI, 0.560 for CTI-BRI, 0.564 for CTI-WHtR, 0.561 for CTI-WWI, and 0.558 for CTI alone. Among these indices, CTI-WHtR showed the numerically highest 4-year AUC, followed by CTI-WWI, CTI-BRI, CTI alone, and CTI-BMI.

### 3.7 Incremental predictive value and comparison with separate-component models

**Figure 5:**
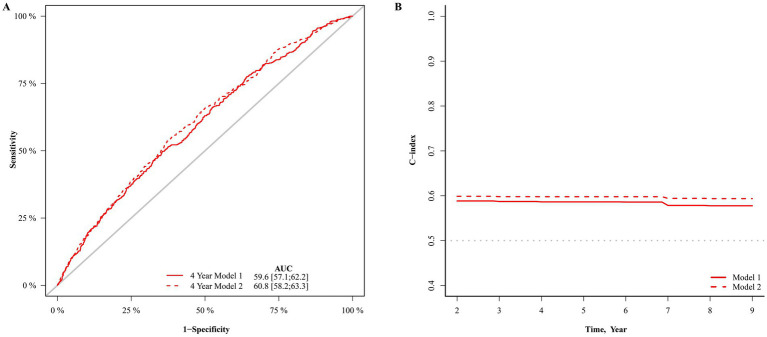
Incremental predictive value of CTI-WHtR beyond the baseline model for incident cardiovascular disease. Incremental predictive performance of CTI-WHtR for incident CVD beyond the baseline model. **(A)** Time-dependent ROC curves at 4 years comparing Model 1 and Model 2. Model 1 represents the baseline model constructed from conventional risk factors, and Model 2 represents the baseline model plus CTI-WHtR. The addition of CTI-WHtR improved the AUC from 59.6 to 60.8%. **(B)** Time-dependent C-index curves for Model 1 and Model 2 during follow-up. Model 2 consistently showed slightly better discriminative ability than Model 1. Abbreviations: CTI-WHtR, C-reactive protein-triglyceride-glucose index × waist-to-height ratio; ROC, receiver operating characteristic; AUC, area under the curve; C-index, concordance index; CVD, cardiovascular disease.

The incremental predictive value of CTI-WHtR beyond the baseline covariate model was evaluated using time-dependent AUC, C-index, NRI, and IDI analyses (Table 3 and Figure 5). Adding CTI-WHtR to the baseline covariate model increased the 4-year AUC from 0.596 (95% CI: 0.571–0.622) to 0.608 (95% CI: 0.582–0.633), corresponding to an absolute increase of 0.012. The NRI was 0.053 (95% CI: 0.009–0.096, *p* = 0.012), and the IDI was 0.005 (95% CI: 0.002–0.010, *p* = 0.004).

**Table 3:** Net reclassification improvement and integrated discrimination improvement analyses of CTI-WHtR indices for the risk of CVD.

Event	NRI (95% CI)	NRI *P*-value	IDI (95% CI)	IDI *P*-value
Baseline model	Ref		Ref	
Baseline model + CTI-WHtR	0.053 (0.009–0.096)	0.012	0.005 (0.002–0.010)	0.004

CVD, cardiovascular disease; CTI-WHtR, C-reactive protein-triglycerides-glucose index - Waist-to-height ratio.

The predictive performance of composite-score models was further compared with that of models including CTI and the corresponding anthropometric indicator as separate predictors (Supplementary Table S6). The baseline model had a 4-year AUC of 0.596 and a Harrell C-index of 0.590. For BMI, both the baseline model plus CTI-BMI and the baseline model plus CTI and BMI had a 4-year AUC of 0.612 and a C-index of 0.609. For BRI, both the baseline model plus CTI-BRI and the baseline model plus CTI and BRI had a 4-year AUC of 0.607, with C-index values of 0.606 and 0.607, respectively. For WHtR, both the baseline model plus CTI-WHtR and the baseline model plus CTI and WHtR had a 4-year AUC of 0.608, with C-index values of 0.605 and 0.606, respectively. For WWI, both the baseline model plus CTI-WWI and the baseline model plus CTI and WWI had a 4-year AUC of 0.601 and a C-index of 0.597.

### 3.8 Sensitivity analyses

Sensitivity analyses are presented in Supplementary Tables S7–S11. After additional adjustment for lipid-lowering and antihypertensive medication use, CTI-WHtR remained associated with incident CVD when modeled as a standardized continuous variable (HR = 1.14, 95% CI: 1.07–1.22, *p* < 0.001). In tertile-based analyses, participants in the highest tertile had a higher risk of incident CVD than those in the lowest tertile (HR = 1.45, 95% CI: 1.23–1.71, *p* < 0.001; Supplementary Table S7). After additional adjustment for antidiabetic medication use, the corresponding HRs were 1.15 (95% CI: 1.08–1.23, *p* < 0.001) for each 1-SD increase and 1.46 (95% CI: 1.25–1.72, *p* < 0.001) for the highest versus lowest tertile (Supplementary Table S8).

After excluding participants with cancer, CTI-WHtR remained associated with incident CVD both as a standardized continuous variable (HR = 1.16, 95% CI: 1.08–1.23, *p* < 0.001) and in tertile-based analyses comparing the highest with the lowest tertile (HR = 1.48, 95% CI: 1.26–1.74, *p* < 0.001; Supplementary Table S9). After excluding participants with liver disease, the corresponding HRs were 1.16 (95% CI: 1.08–1.24, p < 0.001) for each 1-SD increase and 1.48 (95% CI: 1.25–1.74, *p* < 0.001) for the highest versus lowest tertile (Supplementary Table S10).

In the parsimonious clinical risk-factor model, all four CTI-related composite indices remained positively associated with incident CVD when modeled as standardized continuous variables, with HRs ranging from 1.07 for CTI-WWI to 1.17 for CTI-BMI (all *p* ≤ 0.043; Supplementary Table S11). In tertile-based analyses, participants in the highest tertile also had higher CVD risk than those in the lowest tertile across all four indices, with HRs ranging from 1.33 for CTI-WWI to 1.53 for CTI-BMI (all *p* < 0.001).

## 4 Discussion

Based on the nationwide prospective CHARLS cohort, we examined the associations of four CTI-related composite indices—CTI-BMI, CTI-BRI, CTI-WHtR, and CTI-WWI—with incident CVD among Chinese adults aged 45 years and older. Several main findings emerged. First, all four CTI-related composite indices were positively associated with incident CVD after multivariable adjustment, including additional adjustment for hypertension status. Second, restricted cubic spline analyses showed statistically significant nonlinear associations for all four indices, whereas exploratory two-piecewise Cox models did not show clear improvement in model fit over single-line models. Third, CTI-WHtR showed the numerically highest standalone 4-year AUC among the evaluated indices. Fourth, adding CTI-WHtR to the baseline covariate model produced a statistically significant but modest improvement in discrimination and reclassification. Overall, these findings suggest that CTI-related composite adiposity indices, particularly CTI-WHtR, may provide supplementary information for CVD risk stratification, but they should not be interpreted as standalone prediction tools.

Previous studies have predominantly focused on the association of single obesity indicators (e.g., BMI, body fat percentage) or CTI alone with cardiovascular disease (CVD), while comparative studies on the combined assessment of obesity indicators and CTI remain limited (38, 39). One study on patients with acute heart failure only examined the interactions of BMI with BNP and high-sensitivity troponin I, without addressing inflammatory and metabolic indicators such as CTI (40). A separate multicenter study assessed the ability of CTI to predict all-cause and cardiovascular mortality, showing that CTI had independent prognostic significance, although no joint analysis with obesity indicators was performed (41). Furthermore, a prospective cohort study based on CHARLS indicated that the coexistence of high BFP and elevated CTI was linked to a synergistically increased risk of CVD; however, it only focused on a single obesity indicator (BFP) and did not further compare differences when combining CTI with various obesity indicators, including central obesity indices (42).

Unlike the study by Zhou et al. (27), which focused solely on the association between CTI-WHtR and stroke in the CHARLS cohort, our study provides a broader and more comparative evaluation. First, we used a composite cardiovascular endpoint (including both heart disease and stroke), which is more comprehensive for general CVD risk assessment. Second, we systematically compared four CTI-related indices (CTI-BMI, CTI-BRI, CTI-WHtR, and CTI-WWI) within the same framework, whereas their study examined only CTI-WHtR. Thus, our work extends the existing evidence by demonstrating that CTI-WHtR showed the highest standalone AUC among the evaluated CTI-related indices for overall CVD risk stratification.

The positive association observed in this study between CTI-related composite indicators and new-onset CVD risk has a pathophysiological basis. First, CTI reflects the overlap between persistent low-grade inflammatory activity and insulin resistance, both recognized as major contributors to atherosclerosis (43). Insulin resistance can lead to endothelial dysfunction, thereby promoting the development of atherosclerosis (44). Meanwhile, inflammation pervades the entire process of atherogenesis (45). C-reactive protein (CRP), a component of CTI, serves as an inflammatory marker that can directly activate endothelial cells, upregulating adhesion molecule expression and facilitating monocyte infiltration and foam cell formation (46, 47). Elevated triglycerides and fasting glucose reflect insulin resistance, often accompanied by increased free fatty acids, which exacerbate endoplasmic reticulum stress and mitochondrial dysfunction, inducing vascular smooth muscle proliferation and migration (48). Second, obesity, particularly central obesity, further exacerbates inflammatory and metabolic disturbances through the “endocrine-immune” function of adipose tissue. Adipose tissue in obese individuals, especially visceral fat, secretes various adipokines, pro-inflammatory cytokines, and chemokines, collectively contributing to endothelial dysfunction and lipid deposition within the arterial wall (49, 50). Central obesity not only affects vascular function through mechanical compression but also synergistically enhances inflammatory-metabolic stress by releasing pro-inflammatory factors (51–53). Therefore, composite indicators combining CTI and obesity measures may more comprehensively capture the cardiovascular risk arising from the interplay of inflammation, metabolic abnormalities, and atypical fat distribution. Compared with single indicators, CTI combined with obesity indicators can more systematically capture their synergistic roles across the entire course of atherosclerotic development. In this study, the levels of each CTI-related composite indicator were higher in the CVD event group than in the non-event group, and the associations persisted after multivariate adjustment, consistent with the aforementioned potential mechanisms and suggesting that these indicators may supplement risk information not fully captured by traditional risk factors. When CTI and the anthropometric measures were entered into the models separately, CTI remained associated with incident CVD after adjustment for BMI, BRI, WHtR, or WWI. BMI, BRI, and WHtR also retained associations after adjustment for CTI, whereas WWI was attenuated. These findings suggest that the information captured by CTI-BMI, CTI-BRI, and CTI-WHtR is not simply a reflection of either metabolic inflammation or adiposity alone. Rather, these indices appear to represent the overlap between the two risk domains. The prediction analyses also help place these composite indices in context. Models using the multiplicative composite scores showed almost the same 4-year AUC and C-index values as models including CTI and the corresponding anthropometric measure separately. Thus, the composite indices should not be viewed as clearly superior prediction models. Their main practical appeal may be that they condense two related dimensions—metabolic inflammation and adiposity—into a single, easy-to-use marker.

Furthermore, restricted cubic spline analyses showed statistically significant nonlinear associations between all four CTI-related composite indices and incident CVD. Exploratory two-piecewise Cox regression analyses were further performed using the reference points identified from the spline plots. Although positive associations above the reference points were observed for CTI-BRI and CTI-WHtR, the log-likelihood ratio tests did not show statistically significant improvement of the two-piecewise models over the single-line models. Therefore, these reference points should be interpreted as exploratory rather than as definitive clinical thresholds.

The observed nonlinear patterns may reflect the complex relationship between metabolic inflammation, adiposity burden (54), and cardiovascular risk (55). Central adiposity indicators, such as BRI and WHtR, may be particularly relevant because they better capture abdominal fat distribution than BMI, which does not distinguish between fat mass, lean mass, or fat distribution (56). However, given the exploratory nature of the two-piecewise analyses, these findings require confirmation in independent cohorts before specific cutoff values can be proposed.

The multiplicative construction of CTI-related adiposity indices should be interpreted as a pragmatic approach rather than a definitive weighting strategy. This method is simple, reproducible, and consistent with previous CTI-related studies, and it allows metabolic inflammation and adiposity burden to be summarized in a single composite marker. However, multiplication is not the only possible method for integrating these dimensions. Additive combinations, principal component analysis, and weighted scores may capture different information and should be compared in future studies. To reduce the influence of different raw numerical scales, continuous indices were standardized and HRs were reported per 1-SD increase. Therefore, comparisons of continuous associations across indices were based on standardized changes rather than raw-unit changes.

From a risk-prediction perspective, CTI-WHtR showed statistically significant but modest incremental value beyond the baseline model. The absolute improvement in AUC was small, and the NRI and IDI values, although statistically significant, were numerically limited. Therefore, CTI-WHtR should not be interpreted as a standalone prediction tool or as a replacement for established cardiovascular risk assessment models. Rather, because it is derived from routinely available inflammatory, metabolic, and anthropometric measurements, CTI-WHtR may provide supplementary information for population-level risk stratification, particularly in epidemiological or primary prevention settings. Further external validation, calibration assessment, decision-curve analysis, and cost-effectiveness evaluation are needed before its clinical implementation.

From a broader public health perspective, our findings should be interpreted together with evidence on chronic disease prevention and environmental determinants of cardiovascular and cerebrovascular health. In addition to individual-level inflammatory-metabolic and adiposity-related factors, recent studies suggest that health outcomes among middle-aged and older Chinese adults may also be influenced by large-scale chronic disease prevention programs and living environments. For example, evidence from a natural experiment in China indicated that large-scale chronic disease prevention and control programs may improve health outcomes among older adults (57). Another nationwide prospective cohort study reported that living environmental factors were associated with stroke risk in middle-aged and older Chinese adults (58). These studies highlight that CVD prevention in aging populations requires attention to both individual biological risk markers and broader contextual determinants. In this context, CTI-related composite adiposity indices may provide complementary individual-level risk information within population-based chronic disease prevention frameworks.

This investigation possesses several key strengths. By using a large national prospective cohort with prolonged follow-up, it generated evidence that is highly relevant at the population level on CTI-related indices and incident CVD. In addition, we compared four CTI-based composite adiposity markers within the same study and evaluated not only their associations but also their dose–response patterns and predictive performance. Finally, the analyses incorporated multiple complementary statistical approaches, including multivariable Cox models, RCS, time-dependent ROC analysis, and reclassification metrics, and the results were supported by sensitivity analyses.

Nevertheless, several limitations should be acknowledged. First, the analytic sample was restricted to participants with complete information required to calculate the CTI-related composite indices and available follow-up CVD information. Participants excluded from the analytic sample differed from included participants in some baseline characteristics, although most standardized mean differences were small. Therefore, potential selection bias cannot be fully excluded. Multiple imputation was considered but was not used as the primary analytic strategy because the missing data mainly involved key exposure components used to construct the CTI-related indices, and the proportion of missing exposure-related information was substantial. In addition, CHARLS Exit data were linked to the analytic cohort, but no death records were identified in the final complete-case sample; therefore, competing-risk analysis was not performed.

Second, incident CVD was identified using self-reported physician diagnoses of heart disease or stroke rather than objective verification through medical records, electrocardiograms, imaging data, or biomarkers. This approach may have introduced recall bias and outcome misclassification, and asymptomatic, undiagnosed, or unrecognized events may have been missed, leading to potential underestimation of true CVD incidence. In addition, underreporting may differ according to socioeconomic status, health literacy, and access to healthcare, which could introduce differential misclassification and bias the observed associations. Exact dates of CVD onset were also unavailable because incident events were identified at discrete CHARLS follow-up waves; therefore, the Kaplan–Meier curves should be interpreted as wave-based survival plots rather than curves based on continuously recorded event times.

Third, CTI-related composite indices were calculated using baseline measurements only, and changes in inflammatory, metabolic, and adiposity status during follow-up could not be captured. Although multiple covariates were adjusted for, residual confounding from unmeasured or incompletely measured factors, such as diet, physical activity, medication adherence, and long-term blood pressure control, cannot be excluded. In addition, because the multivariable-adjusted model included several sociodemographic, clinical, and laboratory covariates, potential overadjustment cannot be completely ruled out. To address this concern, a parsimonious clinical risk-factor model was fitted as a sensitivity analysis, and the results were generally consistent with the main analyses. Furthermore, because the study population consisted of Chinese adults aged 45 years and older, the generalizability of the findings to younger individuals or other populations requires further validation.

Finally, although CTI-WHtR improved discrimination and reclassification metrics, the magnitude of improvement was modest. Therefore, CTI-WHtR should be interpreted as a supplementary risk marker rather than a standalone clinical screening tool. In addition, the CTI-related composite indices were constructed using a multiplicative form, which is simple and reproducible but remains a pragmatic strategy rather than a definitive weighting method. Future studies should compare multiplicative indices with additive, principal-component-based, or weighted-score approaches and validate their calibration, clinical utility, and cost-effectiveness in independent cohorts.

## 5 Conclusion

In conclusion, CTI-related composite adiposity indices, particularly CTI-WHtR, were independently associated with incident CVD among middle-aged and older Chinese adults. CTI-WHtR provided statistically significant but modest incremental predictive information beyond the baseline risk model. These findings suggest that CTI-WHtR may serve as a potential supplementary marker for CVD risk stratification, but it should not be considered a standalone screening tool. Further studies are warranted to validate its clinical utility, optimal cutoffs, calibration performance, and cost-effectiveness.

## Data Availability

The original contributions presented in the study are included in the article/Supplementary material, further inquiries can be directed to the corresponding author/s.

## References

[1] Global Burden of Cardiovascular Diseases and Risks 2023 Collaborators. Global, regional, and National Burden of cardiovascular diseases and risk factors in 204 countries and territories, 1990-2023. J Am Coll Cardiol. (2025) 86:2167–243. doi: 10.1016/j.jacc.2025.08.015, 40990886

[2] An X Liu Z Zhang L Zhao J Gu Q Han W . Co-occurrence patterns and related risk factors of Ischaemic heart disease and Ischaemic stroke across 203 countries and territories: a spatial correspondence and systematic analysis. Lancet Glob Health. (2025) 13:e808–19. doi: 10.1016/s2214-109x(25)00013-0 40288393

[3] Chong B Jayabaskaran J Jauhari SM Chan SP Goh R Kueh MTW . Global burden of cardiovascular diseases: projections from 2025 to 2050. Eur J Prev Cardiol. (2025) 32:1001–15. doi: 10.1093/eurjpc/zwae281, 39270739

[4] Wei W Chen M Wei J Zheng X. Burden of cardiovascular diseases attributable to diet high in sodium in China and the Global from 1990 to 2021. Curr Probl Cardiol. (2026) 51:103248. doi: 10.1016/j.cpcardiol.2025.103248 41421431

[5] Wang N Salam A Pant R Kumar A Dhurjati R Haghdoost F . Blood pressure-lowering efficacy of antihypertensive drugs and their combinations: a systematic review and Meta-analysis of randomised, double-blind, Placebo-Controlled Trials. Lancet. (2025) 406:915–25. doi: 10.1016/s0140-6736(25)00991-2, 40885583

[6] Zaman S Wasfy JH Kapil V Ziaeian B Parsonage WA Sriswasdi S . The lancet commission on rethinking coronary artery disease: moving from Ischaemia to atheroma. Lancet. (2025) 405:1264–312. doi: 10.1016/s0140-6736(25)00055-8, 40179933 PMC12315672

[7] Khan SS Abdalla M Bello NA Blyler CA Carter J Commodore-Mensah Y . Use of risk assessment to guide decision-making for blood pressure Management in the Primary Prevention of cardiovascular disease: a scientific statement from the American Heart Association and American College of Cardiology. Hypertension. (2025) 82:e317–36. doi: 10.1161/hyp.0000000000000248 40875788

[8] Li X Zhao W Zhao L Sun T Pan H Wang D. Cardiovascular disease risk estimates for primary prevention in the us prediabetes and diabetes population using the Prevent equation. Diabetes Obes Metab. (2025) 27:6470–9. doi: 10.1111/dom.70043 40832797

[9] Khan SS Matsushita K Sang Y Ballew SH Grams ME Surapaneni A . Development and validation of the American Heart Association's Prevent equations. Circulation. (2024) 149:430–49. doi: 10.1161/circulationaha.123.067626, 37947085 PMC10910659

[10] SCORE2 working group and ESC cardiovascular risk collaboration. Score2 risk prediction algorithms: new models to estimate 10-year risk of cardiovascular disease in Europe. Eur Heart J. (2021) 42:2439–54. doi: 10.1093/eurheartj/ehab309, 34120177 PMC8248998

[11] SCORE2-OP working group and ESC cardiovascular risk collaboration. Score2-Op risk prediction algorithms: estimating incident cardiovascular event risk in older persons in four geographical risk regions. Eur Heart J. (2021) 42:2455–67. doi: 10.1093/eurheartj/ehab312, 34120185 PMC8248997

[12] Yang X Li J Hu D Chen J Li Y Huang J . Predicting the 10-year risks of atherosclerotic cardiovascular disease in Chinese population: the China-par project (prediction for Ascvd risk in China). Circulation. (2016) 134:1430–40. doi: 10.1161/circulationaha.116.022367 27682885

[13] Mekhael M Bidaoui G Falloon A Pandey AC. Personalization of primary prevention: exploring the role of coronary artery calcium and polygenic risk score in cardiovascular diseases. Trends Cardiovasc Med. (2025) 35:154–63. doi: 10.1016/j.tcm.2024.10.003, 39442739

[14] Qureshi K Schick J Anderson TR Farooq MU Gorelick PB. Obesity, hypertension and brain health. Curr Hypertens Rep. (2025) 27:24. doi: 10.1007/s11906-025-01343-6 41099969

[15] Xing Y Lin X. Challenges and advances in the Management of Inflammation in atherosclerosis. J Adv Res. (2025) 71:317–35. doi: 10.1016/j.jare.2024.06.016 38909884 PMC12126742

[16] He Y He J Chen D Xiao J. Metabolic score for insulin resistance and the incidence of cardiovascular disease: a Meta-analysis of cohort studies. Front Endocrinol. (2025) 16:1699985. doi: 10.3389/fendo.2025.1699985 PMC12575141 41180178

[17] Yanai H Adachi H Hakoshima M Iida S Katsuyama H. Metabolic-dysfunction-associated steatotic liver disease-its pathophysiology, association with atherosclerosis and cardiovascular disease, and treatments. Int J Mol Sci. (2023) 24:15473. doi: 10.3390/ijms242015473, 37895151 PMC10607514

[18] Sun Y Guo Y Ma S Mao Z Meng D Xuan K . Association of C-reactive protein-triglyceride glucose index with the incidence and mortality of cardiovascular disease: a retrospective cohort study. Cardiovasc Diabetol. (2025) 24:313. doi: 10.1186/s12933-025-02835-0, 40750895 PMC12317521

[19] Ma X Ma X Wang Y Qiu G Zhang C. Associations of cumulative exposure and dynamic trajectories of the C-reactive protein-triglyceride-glucose index with incident cardiovascular disease in middle-aged and older Chinese adults: a Nationwide cohort study. Cardiovasc Diabetol. (2025) 24:303. doi: 10.1186/s12933-025-02869-4, 40713770 PMC12296596

[20] Tang N Chen X Li H Cheng S Hu Y Wang L . Association of C reactive protein triglyceride glucose index with mortality in coronary heart disease and type 2 diabetes from Nhanes data. Sci Rep. (2025) 15:24687. doi: 10.1038/s41598-025-10184-x 40634472 PMC12241523

[21] Zhou Y Lin H Weng X Dai H Xu J. Correlation between Hs-Crp-triglyceride glucose index and Nafld and liver fibrosis. BMC Gastroenterol. (2025) 25:252. doi: 10.1186/s12876-025-03870-7, 40221654 PMC11994022

[22] Valenzuela PL Carrera-Bastos P Castillo-García A Lieberman DE Santos-Lozano A Lucia A. Obesity and the risk of Cardiometabolic diseases. Nat Rev Cardiol. (2023) 20:475–94. Epub 2023/03/18. doi: 10.1038/s41569-023-00847-5, 36927772

[23] Calderón-García JF Roncero-Martín R Rico-Martín S De Nicolás-Jiménez JM López-Espuela F Santano-Mogena E . Effectiveness of body roundness index (Bri) and a body shape index (Absi) in predicting hypertension: a systematic review and meta-analysis of observational studies. Int J Environ Res Public Health. (2021) 18:11607. doi: 10.3390/ijerph182111607, 34770120 PMC8582804

[24] Zhang X Lu X Pan X Shen S Tong N. Role of waist circumference-to-height ratio in assessing adiposity, predicting type 2 diabetes mellitus and other Cardiometabolic diseases. Zhong Nan Da Xue Xue Bao Yi Xue Ban. (2024) 49:1062–72. doi: 10.11817/j.issn.1672-7347.2024.240259, 39788494 PMC11495984

[25] Zhao Y Hu Y Smith JP Strauss J Yang G. Cohort profile: the China health and retirement longitudinal study (Charls). Int J Epidemiol. (2012) 43:61–8. doi: 10.1093/ije/dys203, 23243115 PMC3937970

[26] Du J Man Z Bai X Luo D Guo Q Wang Y . Association of C-reactive protein-triglycerides-glucose index-waist to height ratio and cardiometabolic multimorbidity in middle-aged and older adults: a nationwide cohort study. Cardiovasc Diabetol. (2026) 25:122. doi: 10.1186/s12933-026-03151-x 41923235 PMC13067722

[27] Zhou Z Yang J Hou J Zhang L. The predictive value of C-reactive protein-triglycerides-glucose index-waist-to-height ratio for stroke: a Nationwide cohort study. Cardiovasc Diabetol. (2026) 25:18. doi: 10.1186/s12933-025-03065-0, 41547799 PMC12817842

[28] Huo G Tang Y Liu Z Cao J Yao Z Zhou D. Association between C-reactive protein-triglyceride glucose index and stroke risk in different glycemic status: insights from the China health and retirement longitudinal study (Charls). Cardiovasc Diabetol. (2025) 24:142. doi: 10.1186/s12933-025-02686-9, 40140859 PMC11948880

[29] Yang M Liu J Shen Q Chen H Liu Y Wang N . Body roundness index trajectories and the incidence of cardiovascular disease: evidence from the China health and retirement longitudinal study. J Am Heart Assoc. (2024) 13:e034768. doi: 10.1161/JAHA.124.034768, 39319466 PMC11681446

[30] Liu H Zhi J Zhang C Huang S Ma Y Luo D . Association between weight-adjusted waist index and depressive symptoms: a nationally representative cross-sectional study from Nhanes 2005 to 2018. J Affect Disord. (2024) 350:49–57. doi: 10.1016/j.jad.2024.01.104, 38220117

[31] Thomas DM Bredlau C Bosy-Westphal A Mueller M Shen W Gallagher D . Relationships between body roundness with body fat and visceral adipose tissue emerging from a new geometrical model. Obesity (Silver Spring). (2013) 21:2264–71. doi: 10.1002/oby.20408, 23519954 PMC3692604

[32] Feng Q Bešević J Conroy M Omiyale W Woodward M Lacey B . Waist-to-height ratio and body fat percentage as risk factors for ischemic cardiovascular disease: a prospective cohort study from Uk biobank. Am J Clin Nutr. (2024) 119:1386–96. doi: 10.1016/j.ajcnut.2024.03.018, 38839194 PMC11196863

[33] Zheng W Man Z Li Y Zhu X. Association between the triglyceride glucose index:Chinese visceral adiposity index (Tyg-Cvai) and new-onset cardiovascular disease in middle-aged and older adults-insights from the China health and retirement longitudinal study (Charls). Cardiovasc Diabetol. (2026) 25:48. doi: 10.1186/s12933-025-03063-2, 41559650 PMC12903522

[34] Gao K Cao L-F Ma W-Z Gao Y-J Luo M-S Zhu J . Association between sarcopenia and cardiovascular disease among middle-aged and older adults: findings from the China health and retirement longitudinal study. EClinicalMedicine. (2022) 44:101264. doi: 10.1016/j.eclinm.2021.101264 35059617 PMC8760427

[35] Gan YY Luo YD Zhai L Huo RR Dai X Liao Q. Temporal trends, associated risk factors and longitudinal cardiovascular outcomes of body roundness among middle-aged and older Chinese adults: from the China health and retirement longitudinal study 2011-2018. Front Nutr. (2025) 12:1515067. doi: 10.3389/fnut.2025.1515067, 39927280 PMC11804525

[36] Huo G Tan Z Tang Y Huang J Cao J Zhou D. Association between triglyceride glucose weight adjusted waist index and stroke risk in different glucose metabolism status. Sci Rep. (2025) 15:15813. doi: 10.1038/s41598-025-99618-0, 40328907 PMC12056119

[37] Wang Z Zhu J Xuan S Dong S Shen Z Chen S . Associations of estimated glucose disposal rate with frailty progression: results from two prospective cohorts. Cardiovasc Diabetol. (2025) 24:81. doi: 10.1186/s12933-025-02650-7, 39972476 PMC11841016

[38] Wang C He S Xie G Zhang S Xiong Z Lu H . Associations of longitudinal trajectories of triglyceride-glucose index combined with classical and novel obesity indices and cardiovascular disease: evidence from a Nationwide prospective cohort study in China. Cardiovasc Diabetol. (2025) 24:431. doi: 10.1186/s12933-025-02972-6, 41225604 PMC12613409

[39] Wang L Ding H Deng Y Huang J Lao X Wong MCS. Associations of obesity indices change with cardiovascular outcomes: a dose-response Meta-analysis. Int J Obes. (2024) 48:635–45. doi: 10.1038/s41366-024-01485-8, 38336864

[40] Horiuchi YU Wettersten N Vanveldhuisen DJ Mueller C Nowak R Hogan C . The influence of body mass index on clinical interpretation of established and novel biomarkers in acute heart failure. J Card Fail. (2023) 29:1121–31. doi: 10.1016/j.cardfail.2023.03.029 37127240 PMC11436290

[41] Wang M Qiu W Chu R Li L Xu Q Wen Y . Prognostic significance of the C-reactive protein-triglyceride-glucose index for all-cause and cardiovascular mortality in peritoneal dialysis: a multicenter cohort study. Sci Rep. (2025) 16:2510. doi: 10.1038/s41598-025-32217-1, 41444370 PMC12820109

[42] Tian X Qu Z Yang X Cao Y Zhang B. Synergistic effects of body fat percentage and C-reactive protein triglyceride-glucose index on cardiovascular disease risk: a Chinese cohort study. Sci Rep. (2025) 15:40266. doi: 10.1038/s41598-025-24094-5, 41249375 PMC12624090

[43] Brie AD Christodorescu RM Popescu R Adam O Tîrziu A Brie DM. Atherosclerosis and insulin resistance: is there a link between them? Biomedicine. (2025) 13:1291. doi: 10.3390/biomedicines13061291, 40564010 PMC12189823

[44] Ashraf FUN Ghouri K Someshwar F Kumar S Kumar N Kumari K . Insulin resistance and coronary artery disease: untangling the web of endocrine-cardiac connections. Cureus. (2023) 15:e51066. doi: 10.7759/cureus.51066 38269234 PMC10806385

[45] Libby P Soehnlein O. Inflammation in atherosclerosis: lessons and therapeutic implications. Immunity. (2025) 58:2383–401. doi: 10.1016/j.immuni.2025.09.012, 41045921

[46] Attiq A Afzal S Ahmad W Kandeel M. Hegemony of inflammation in atherosclerosis and coronary artery disease. Eur J Pharmacol. (2024) 966:176338. doi: 10.1016/j.ejphar.2024.176338, 38242225

[47] Badimon L Peña E Arderiu G Padró T Slevin M Vilahur G . C-reactive protein in atherothrombosis and angiogenesis. Front Immunol. (2018) 9:430. doi: 10.3389/fimmu.2018.00430, 29552019 PMC5840191

[48] Poznyak A Grechko AV Poggio P Myasoedova VA Alfieri V Orekhov AN. The diabetes mellitus-atherosclerosis connection: the role of lipid and glucose metabolism and chronic inflammation. Int J Mol Sci. (2020) 21:1835. doi: 10.3390/ijms21051835, 32155866 PMC7084712

[49] Banerjee D Mani A. Obesity's systemic impact: exploring molecular and physiological links to diabetes, cardiovascular disease, and heart failure. Front Endocrinol (Lausanne). (2025) 16:1681766. doi: 10.3389/fendo.2025.1681766, 41282294 PMC12634369

[50] Raman P Khanal S. Leptin in atherosclerosis: focus on macrophages, endothelial and smooth muscle cells. Int J Mol Sci. (2021) 22:5446. doi: 10.3390/ijms22115446, 34064112 PMC8196747

[51] Młynarska E Bojdo K Frankenstein H Krawiranda K Kustosik N Lisińska W . Endothelial dysfunction as the common pathway linking obesity, hypertension and atherosclerosis. Int J Mol Sci. (2025) 26:10096. doi: 10.3390/ijms262010096, 41155389 PMC12564390

[52] Laskou M Delbare S Gildea M Weinstock A Moura Virginio V La Forest M . Platelets impair the resolution of inflammation in atherosclerotic plaques in insulin-resistant mice after lipid lowering. JCI Insight. (2025) 10:e193593. doi: 10.1172/jci.insight.193593, 41066197 PMC12643486

[53] Donath MY Drucker DJ. Obesity, diabetes, and inflammation: pathophysiology and clinical implications. Immunity. (2025) 58:2373–82. doi: 10.1016/j.immuni.2025.09.011, 41045922

[54] Cheong LYT Saipuljumri EN Loi GWZ Zeng J Lo CH. Autolysosomal dysfunction in obesity-induced metabolic inflammation and related disorders. Curr Obes Rep. (2025) 14:43. doi: 10.1007/s13679-025-00638-8, 40366502 PMC12078456

[55] Pasut A Lama E Van Craenenbroeck AH Kroon J Carmeliet P. Endothelial cell metabolism in cardiovascular physiology and disease. Nat Rev Cardiol. (2025) 22:923–43. doi: 10.1038/s41569-025-01162-x, 40346347

[56] Kee CC Sumarni MG Lim KH Selvarajah S Haniff J Tee GHH . Association of Bmi with risk of Cvd mortality and all-cause mortality. Public Health Nutr. (2017) 20:1226–34. doi: 10.1017/s136898001600344x, 28077198 PMC10261502

[57] Ma Q Han Y Chen M Hu F Zhou H. The impact of a large-scale chronic disease prevention and control program on the health benefits of older adults: evidence from a natural experiment in China. China Econ Rev. (2026) 95:102632. doi: 10.1016/j.chieco.2025.102632

[58] Huang X Yang B Liu N Jiang X Lin Q Gao W . Association between living environmental factors and stroke in middle-aged and older Chinese adults: a Nationwide prospective cohort study. J Am Heart Assoc. (2026) 15:e043867. doi: 10.1161/jaha.125.043867, 41614318 PMC13055457

