# Supplementary material for: CTI-related composite adiposity indices and future cardiovascular disease risk among middle-aged and older Chinese adults
Source: Front Nutr. 2026 Jun 4;13:1849538. doi: 10.3389/fnut.2026.1849538 (PMC13275352; doi:10.3389/fnut.2026.1849538)
Supplement: Supplementary file 1 [file Table_1.docx]

Supplementary Material

**Table S1.** Baseline characteristics of included participants and participants excluded at the final step

**Table S2.** Assessment of multicollinearity across covariates.

**Table S3.** Schoenfeld residual–based tests of the proportional hazards assumption for the fully adjusted Cox model (overall follow-up).

**Table S4.** Mutually adjusted associations of CTI and anthropometric indices with incident CVD

**Table S5.** Exploratory two-piecewise Cox regression analyses of CTI-related composite indices and incident CVD risk.

**Table S6.** Predictive performance of composite-score models and separate-component models for incident CVD

**Table S7.** Associations between CTI-related indicators and the risk of CVD after adding patients receiving lipid-lowering drugs or hypoglycemic therapy.

**Table S8.** Associations between CTI-related indicators and the risk of CVD after adding patients taking prescription for diabetes.

**Table S9.** Associations between CTI-related indicators and the risk of CVD after exclude cancer participants.

**Table S10.** Associations between CTI-related indicators and the risk of CVD after exclude liver disease participants.

| **Table S1.** Baseline characteristics of included participants and participants excluded at the final step | | | | |
| --- | --- | --- | --- | --- |
| Characteristic | Included (N=5290) | Excluded (N=4262) | P value | SMD |
| Age | 58.06 ± 8.51 (n=5290) | 56.44 ± 9.20 (n=4261) | <0.001 | 0.183 |
| TC, mg/dL | 194.89 ± 38.77 (n=5290) | 187.35 ± 37.17 (n=1623) | <0.001 | 0.199 |
| HDL-C, mg/dL | 51.77 ± 15.20 (n=5290) | 49.92 ± 15.26 (n=1628) | <0.001 | 0.122 |
| TG, mg/dL | 101.78 (72.57, 147.79) (n=5290) | 110.62 (78.76, 168.15) (n=1624) | <0.001 | 0.105 |
| Scr, mg/dL | 0.77 ± 0.18 (n=5287) | 0.76 ± 0.17 (n=1616) | 0.164 | 0.039 |
| Cystatin C, mg/dL | 0.99 ± 0.23 (n=3934) | 0.95 ± 0.22 (n=1164) | <0.001 | 0.177 |
| UA, mg/dL | 4.36 ± 1.20 (n=5290) | 4.39 ± 1.21 (n=1627) | 0.406 | 0.024 |
| HbA1c | 5.26 ± 0.77 (n=5259) | 5.14 ± 0.76 (n=1708) | <0.001 | 0.165 |
| FBG, mg/dL | 108.48 ± 32.42 (n=5290) | 107.89 ± 31.19 (n=942) | 0.593 | 0.019 |
| BMI | 23.52 ± 3.83 (n=5290) | 23.41 ± 3.51 (n=830) | 0.448 | 0.027 |
| BRI | 4.13 ± 1.48 (n=5290) | 4.08 ± 1.45 (n=839) | 0.386 | 0.032 |
| WHtR | 0.53 ± 0.08 (n=5290) | 0.53 ± 0.08 (n=839) | 0.400 | 0.031 |
| WWI | 11.03 ± 1.29 (n=5290) | 10.98 ± 1.34 (n=810) | 0.396 | 0.032 |
| Sex |  |  | <0.001 | 0.079 |
| Female | 2909 (54.99) | 2172 (51.03) |  |  |
| Male | 2381 (45.01) | 2084 (48.97) |  |  |
| Ethnicity |  |  | <0.001 | 0.089 |
| Others | 326 (6.16) | 361 (8.47) |  |  |
| Han | 4964 (93.84) | 3901 (91.53) |  |  |
| Marital status |  |  | 0.534 | 0.013 |
| Unmarried | 500 (9.45) | 387 (9.08) |  |  |
| Married | 4790 (90.55) | 3875 (90.92) |  |  |
| Education |  |  | <0.001 | 0.105 |
| Below primary school | 3702 (69.98) | 2768 (65.05) |  |  |
| Junior school and above | 1588 (30.02) | 1487 (34.95) |  |  |
| Residence place |  |  | 0.015 | 0.050 |
| Rural | 3625 (68.53) | 2821 (66.19) |  |  |
| Urban | 1665 (31.47) | 1441 (33.81) |  |  |
| Smoking status |  |  | <0.001 | 0.143 |
| Never | 3314 (62.65) | 2583 (60.65) |  |  |
| Former | 401 (7.58) | 502 (11.79) |  |  |
| Current | 1575 (29.77) | 1174 (27.57) |  |  |
| Alcohol consumption |  |  | 0.559 | 0.012 |
| No | 3491 (65.99) | 2785 (65.42) |  |  |
| Yes | 1799 (34.01) | 1472 (34.58) |  |  |
| Hypertension status |  |  | <0.001 | 0.091 |
| No | 4164 (79.15) | 3503 (82.72) |  |  |
| Yes | 1097 (20.85) | 732 (17.28) |  |  |
| Diabetes |  |  | 0.053 | 0.040 |
| No | 5007 (95.63) | 4083 (96.41) |  |  |
| Yes | 229 (4.37) | 152 (3.59) |  |  |
| Dyslipidemia |  |  | 0.040 | 0.043 |
| No | 4794 (92.64) | 3925 (93.72) |  |  |
| Yes | 381 (7.36) | 263 (6.28) |  |  |
| Liver disease |  |  | 0.801 | 0.005 |
| No | 5148 (97.32) | 4144 (97.23) |  |  |
| Yes | 142 (2.68) | 118 (2.77) |  |  |
| Cancer |  |  | 0.790 | 0.005 |
| No | 5258 (99.40) | 4238 (99.44) |  |  |
| Yes | 32 (0.60) | 24 (0.56) |  |  |
| Taking prescription for cholesterol |  |  | 0.042 | 0.042 |
| No | 5077 (95.97) | 4124 (96.76) |  |  |
| Yes | 213 (4.03) | 138 (3.24) |  |  |
| Taking prescription for hypertension |  |  | <0.001 | 0.083 |
| No | 4481 (84.71) | 3733 (87.59) |  |  |
| Yes | 809 (15.29) | 529 (12.41) |  |  |
| Taking prescription for diabetes |  |  | 0.547 | 0.012 |
| No | 5148 (97.32) | 4156 (97.51) |  |  |
| Yes | 142 (2.68) | 106 (2.49) |  |  |
| Values are presented as mean ± SD, median (IQR), or n (%), as appropriate. Percentages were calculated among non-missing observations. P values were calculated using Welch's t test for normally distributed continuous variables, the Mann-Whitney U test for skewed continuous variables, and the chi-square test for categorical variables. SMD denotes standardized mean difference; for categorical variables, the maximum absolute category-specific SMD is reported. Excluded participants refer to the 4,262 individuals removed at the final step of sample selection because CTI-related composite indices could not be calculated and/or age was <45 years. | | | | |
|  |  |  |  |  |
|  |  |  |  |  |
|  |  |  |  |  |
|  |  |  |  |  |
|  |  |  |  |  |
|  |  |  |  |  |

| **Table S2.** Assessment of multicollinearity across covariates. | | | | |
| --- | --- | --- | --- | --- |
| Term1 | GVIF | DF | GVIF^(1/(2*Df)) | Colinearity (0=No, 1=Yes) |
| Crude | 1.512 | 1 | 1.229 | 0 |
| TC_mg.dl | 1.175 | 1 | 1.084 | 0 |
| HDL_md.dl | 1.358 | 1 | 1.165 | 0 |
| sex | 2.848 | 1 | 1.688 | 0 |
| age | 1.257 | 1 | 1.121 | 0 |
| marital_status | 1.102 | 1 | 1.050 | 0 |
| education | 1.232 | 1 | 1.110 | 0 |
| residence_place | 1.065 | 1 | 1.032 | 0 |
| nationality.han | 1.015 | 1 | 1.008 | 0 |
| smoke | 2.117 | 2 | 1.206 | 0 |
| drink | 1.385 | 1 | 1.177 | 0 |
| HbA1c | 1.100 | 1 | 1.049 | 0 |
| creatinine | 1.619 | 1 | 1.272 | 0 |
| UA | 1.541 | 1 | 1.241 | 0 |
| cancer | 1.003 | 1 | 1.002 | 0 |
| liver.disease | 1.009 | 1 | 1.004 | 0 |
| Abbreviations: CVD, cardiovascular disease; BMI, body mass index; HDL-C, high-density lipoprotein cholesterol; TG, triglyceride; TC, total cholesterol; FBG, fasting blood glucose; HbA1c, hemoglobin A1c; WC, waist circumference; UA, uric acid; Scr, serum creatinine; CTI, C-reactive protein-triglycerides-glucose index; CTI-WHtR, C-reactive protein-triglycerides-glucose index - Waist-to-height ratio; CTI-BMI, C-reactive protein-triglycerides-glucose index - Body mass index; CTI-WWI, C-reactive protein-triglycerides-glucose index - Waist-to-weight index; CTI-BRI, C-reactive protein-triglycerides-glucose index - Body roundness index. | | | | |
|  |  |  |  |  |
|  |  |  |  |  |
|  |  |  |  |  |
|  |  |  |  |  |
|  |  |  |  |  |
|  |  |  |  |  |
|  |  |  |  |  |
|  |  |  |  |  |

| **Table S3.** Schoenfeld residual–based tests of the proportional hazards assumption for the fully adjusted Cox model (overall follow-up). | | | |
| --- | --- | --- | --- |
|  |  |  |  |
| **Variable** | **chisq** | **df** | **p.value** |
| CTIWHtR | 0.032 | 1 | 0.859 |
| CTIBRI | 0.033 | 1 | 0.856 |
| CTIABSI | 0.063 | 1 | 0.801 |
| CTIWWI | 0.012 | 1 | 0.913 |
| sex | 0.106 | 1 | 0.745 |
| age | 0.369 | 1 | 0.543 |
| marital_status | 0.619 | 1 | 0.431 |
| education | 4.002 | 1 | 0.045 |
| residence_place | 0.835 | 1 | 0.361 |
| nationality.han | 2.197 | 1 | 0.138 |
| smoke | 0.009 | 1 | 0.924 |
| drink | 1.853 | 1 | 0.173 |
| HbA1c | 1.754 | 1 | 0.185 |
| UA | 1.261 | 1 | 0.261 |
| creatinine | 0.143 | 1 | 0.706 |
| cancer | 1.318 | 1 | 0.251 |
| liver.disease | 0.209 | 1 | 0.647 |
| TC_mg.dl | 1.461 | 1 | 0.227 |
| HDL_md.dl | 0.124 | 1 | 0.725 |
| GLOBAL | 19.189 | 20 | 0.510 |
| Abbreviations: CVD, cardiovascular disease; BMI, body mass index; HDL-C, high-density lipoprotein cholesterol; TG, triglyceride; TC, total cholesterol; FBG, fasting blood glucose; HbA1c, hemoglobin A1c; WC, waist circumference; UA, uric acid; Scr, serum creatinine; CTI, C-reactive protein-triglycerides-glucose index; CTI-WHtR, C-reactive protein-triglycerides-glucose index - Waist-to-height ratio; CTI-BMI, C-reactive protein-triglycerides-glucose index - Body mass index; CTI-WWI, C-reactive protein-triglycerides-glucose index - Waist-to-weight index; CTI-BRI, C-reactive protein-triglycerides-glucose index - Body roundness index. | | | |
|  |  |  |  |
|  |  |  |  |
|  |  |  |  |
|  |  |  |  |
|  |  |  |  |
|  |  |  |  |
|  |  |  |  |
|  |  |  |  |
|  |  |  |  |

| **Table S4.** Mutually adjusted associations of CTI and anthropometric indices with incident CVD | | | | |
| --- | --- | --- | --- | --- |
| **Pair** | **Single-component model, HR (95% CI)** | **P value** | **Mutually adjusted model, HR (95% CI)** | **P value** |
| CTI + BMI |  |  |  |  |
| CTI, per 1-SD increase | 1.10 (1.03, 1.17) | 0.003 | 1.08 (1.01, 1.15) | 0.022 |
| BMI, per 1-SD increase | 1.17 (1.11, 1.23) | <0.001 | 1.16 (1.10, 1.22) | <0.001 |
| CTI + BRI |  |  |  |  |
| CTI, per 1-SD increase | 1.10 (1.03, 1.17) | 0.003 | 1.09 (1.02, 1.16) | 0.011 |
| BRI, per 1-SD increase | 1.13 (1.06, 1.20) | <0.001 | 1.12 (1.05, 1.19) | <0.001 |
| CTI + WHtR |  |  |  |  |
| CTI, per 1-SD increase | 1.10 (1.03, 1.17) | 0.003 | 1.09 (1.02, 1.16) | 0.009 |
| WHtR, per 1-SD increase | 1.12 (1.05, 1.19) | <0.001 | 1.11 (1.04, 1.18) | 0.001 |
| CTI + WWI |  |  |  |  |
| CTI, per 1-SD increase | 1.10 (1.03, 1.17) | 0.003 | 1.10 (1.03, 1.17) | 0.003 |
| WWI, per 1-SD increase | 1.02 (0.96, 1.09) | 0.45 | 1.02 (0.96, 1.08) | 0.489 |
| Notes: Single-component models included either CTI or the corresponding anthropometric index, together with covariates. Mutually adjusted models included CTI and the corresponding anthropometric index simultaneously as separate predictors. Continuous variables were standardized, and HRs were reported per 1-SD increase. Models were adjusted for sex, age, smoking status, alcohol drinking, marital status, education, residence place, ethnicity, cancer, liver disease, TC, HDL-C, UA, HbA1c, Scr, and hypertension status. | | | | |
|  |  |  |  |  |
|  |  |  |  |  |
|  |  |  |  |  |
|  |  |  |  |  |

| **Table S5.** Exploratory two-piecewise Cox regression analyses of CTI-related composite indices and incident CVD risk. | | |
| --- | --- | --- |
|  |  |  |
| Outcome: | CVD | |
|  | Adjusted HR(95%CI) | *P-*value |
| Inflection point of CTI-BMI | 107.360 | |
| CTI-BMI < 107.360 | 1.010 (1.001,1.017) | 0.029 |
| CTI-BMI ≥ 107.360 | 1.006 (1.002,1.010) | 0.006 |
| *P* for log-likelihood ratio test | 0.533 | |
| Inflection point of CTI-BRI | 18.610 | |
| CTI-BRI < 18.610 | 1.002 (0.982,1.022) | 0.862 |
| CTI-BRI ≥ 18.610 | 1.023 (1.010,1.037) | < 0.001 |
| *P* for log-likelihood ratio test | 0.170 | |
| Inflection point of CTI-WHtR | 2.481 | |
| CTI-WHtR < 2.481 | 1.061 (0.808,1.395) | 0.669 |
| CTI-WHtR ≥ 2.481 | 1.262 (1.010,1.577) | 0.041 |
| *P* for log-likelihood ratio test | 0.524 | |
| Inflection point of CTI-WWI | 51.65 | |
| CTI-WWI < 51.650 | 1.000 (0.988,1.014) | 0.634 |
| CTI-WWI ≥ 51.650 | 1.001 (0.987,1.015) | 0.879 |
| *P* for log-likelihood ratio test | 0.737 | |
| Models were adjusted for sex, age, smoking status, alcohol drinking, marital status, education, residence place, race, cancer, liver disease, TC, HDL-C, UA, HbA1c, Scr, and hypertension status. The identified points were consistent with the vertical dashed lines shown in Figure 3. Because the log-likelihood ratio tests were not statistically significant, these points should be interpreted as exploratory turning points rather than definitive thresholds. | | |
|  |  |  |
|  |  |  |
|  |  |  |
|  |  |  |
|  |  |  |
|  |  |  |

| **Table S6.** Predictive performance of composite-score models and separate-component models for incident CVD | | | |
| --- | --- | --- | --- |
| **Model type** | **Predictors added to baseline model** | **4-year AUC** | **Harrell C-index** |
| Baseline model | Covariates only | 0.596 | 0.59 |
| Composite-score model | CTI-BMI | 0.612 | 0.609 |
| Separate-component model | CTI + BMI | 0.612 | 0.609 |
| Composite-score model | CTI-BRI | 0.607 | 0.606 |
| Separate-component model | CTI + BRI | 0.607 | 0.607 |
| Composite-score model | CTI-WHtR | 0.608 | 0.605 |
| Separate-component model | CTI + WHtR | 0.608 | 0.606 |
| Composite-score model | CTI-WWI | 0.601 | 0.597 |
| Separate-component model | CTI + WWI | 0.601 | 0.597 |
| Composite-score models included the baseline model plus one CTI-related composite index. Separate-component models included the baseline model plus CTI and the corresponding anthropometric index as separate predictors. The baseline model included sex, age, smoking status, alcohol drinking, marital status, education, residence place, ethnicity, cancer, liver disease, TC, HDL-C, UA, HbA1c, and Scr. Predictive performance was assessed using 4-year AUC and Harrell C-index. | | | |
|  |  |  |  |
|  |  |  |  |
|  |  |  |  |
|  |  |  |  |
|  |  |  |  |
|  |  |  |  |

| **Table S7.** Associations between CTI-related indicators and the risk of CVD after adding patients receiving lipid-lowering drugs or hypoglycemic therapy. | | | | | | | | |
| --- | --- | --- | --- | --- | --- | --- | --- | --- |
|  |  |  |  |  |  |  |  |  |
|  | Model 1 | |  | Model 2 | |  | Model 3 | |
|  | HR (95% CI) | *P* value |  | HR (95% CI) | *P* value |  | HR (95% CI) | *P* value |
| **CTI-WHtR, per 1-SD increase** | 1.26 (1.20~1.33) | <0.001 |  | 1.24 (1.17~1.30) | <0.001 |  | 1.14 (1.07~1.22) | <0.001 |
| **CTI-WHtR terciles** |  |  |  |  |  |  |  |  |
| T1 | 1.0 [Ref] | |  | 1.0 [Ref] | |  | 1.0 [Ref] | |
| T2 | 1.38 (1.20~1.59) | <0.001 |  | 1.34 (1.16~1.54) | <0.001 |  | 1.28 (1.10~1.48) | 0.001 |
| T3 | 1.79 (1.56~2.04) | <0.001 |  | 1.70 (1.48~1.96) | <0.001 |  | 1.45 (1.23~1.71) | <0.001 |
| *P-*trend | 1.33 (1.25~1.42) | <0.001 |  | 1.30 (1.22~1.39) | <0.001 |  | 1.20 (1.11~1.30) | <0.001 |
| Model 1: no covariates were adjusted Model 2: Sex, Age, Smoking, Drinking were adjusted Model 3: Fully adjusted for variables, including Sex, Age, Smoking, Drinking, Marital status, Education, Residence place, Race, Cancer, Liver disease, TC, HDL, UA, HbA1c, Scr, Hypertension status, taking prescription for cholesterol, taking prescription for hypertension. Abbreviations: CVD, cardiovascular disease; BMI, body mass index; HDL-C, high-density lipoprotein cholesterol; TG, triglyceride; TC, total cholesterol; FBG, fasting blood glucose; HbA1c, hemoglobin A1c; WC, waist circumference; UA, uric acid; Scr, serum creatinine; CTI, C-reactive protein-triglycerides-glucose index; CTI-WHtR, C-reactive protein-triglycerides-glucose index - Waist-to-height ratio; CTI-BMI, C-reactive protein-triglycerides-glucose index - Body mass index; CTI-WWI, C-reactive protein-triglycerides-glucose index - Waist-to-weight index; CTI-BRI, C-reactive protein-triglycerides-glucose index - Body roundness index. | | | | | | | | |
|  |  |  |  |  |  |  |  |  |
|  |  |  |  |  |  |  |  |  |
|  |  |  |  |  |  |  |  |  |
|  |  |  |  |  |  |  |  |  |
|  |  |  |  |  |  |  |  |  |
|  |  |  |  |  |  |  |  |  |
|  |  |  |  |  |  |  |  |  |
|  |  |  |  |  |  |  |  |  |
|  |  |  |  |  |  |  |  |  |
|  |  |  |  |  |  |  |  |  |
|  |  |  |  |  |  |  |  |  |

| **Table S8.** Associations between CTI-related indicators and the risk of CVD after adding patients taking prescription for diabetes. | | | | | | | | |
| --- | --- | --- | --- | --- | --- | --- | --- | --- |
|  |  |  |  |  |  |  |  |  |
|  | Model 1 | |  | Model 2 | |  | Model 3 | |
|  | HR (95% CI) | *P* value |  | HR (95% CI) | *P* value |  | HR (95% CI) | *P* value |
| **CTI-WHtR, per 1-SD increase** | 1.26 (1.20~1.33) | <0.001 |  | 1.24 (1.17~1.30) | <0.001 |  | 1.15 (1.08~1.23) | <0.001 |
| **CTI-WHtR terciles** |  |  |  |  |  |  |  |  |
| T1 | 1.0 [Ref] | |  | 1.0 [Ref] | |  | 1.0 [Ref] | |
| T2 | 1.38 (1.20~1.59) | <0.001 |  | 1.34 (1.16~1.54) | <0.001 |  | 1.27 (1.10~1.48) | 0.001 |
| T3 | 1.79 (1.56~2.04) | <0.001 |  | 1.70 (1.48~1.96) | <0.001 |  | 1.46 (1.25~1.72) | <0.001 |
| *P-*trend | 1.33 (1.25~1.42) | <0.001 |  | 1.30 (1.22~1.39) | <0.001 |  | 1.21 (1.11~1.31) | <0.001 |
| Model 1: no covariates were adjusted Model 2: Sex, Age, Smoking, Drinking were adjusted Model 3: Fully adjusted for variables, including Sex, Age, Smoking, Drinking, Marital status, Education, Residence place, Race, Cancer, Liver disease, TC, HDL, UA, HbA1c, Scr, Hypertension status, taking prescription for diabetes. Abbreviations: CVD, cardiovascular disease; BMI, body mass index; HDL-C, high-density lipoprotein cholesterol; TG, triglyceride; TC, total cholesterol; FBG, fasting blood glucose; HbA1c, hemoglobin A1c; WC, waist circumference; UA, uric acid; Scr, serum creatinine; CTI, C-reactive protein-triglycerides-glucose index; CTI-WHtR, C-reactive protein-triglycerides-glucose index - Waist-to-height ratio; CTI-BMI, C-reactive protein-triglycerides-glucose index - Body mass index; CTI-WWI, C-reactive protein-triglycerides-glucose index - Waist-to-weight index; CTI-BRI, C-reactive protein-triglycerides-glucose index - Body roundness index. | | | | | | | | |
|  |  |  |  |  |  |  |  |  |
|  |  |  |  |  |  |  |  |  |
|  |  |  |  |  |  |  |  |  |
|  |  |  |  |  |  |  |  |  |
|  |  |  |  |  |  |  |  |  |
|  |  |  |  |  |  |  |  |  |
|  |  |  |  |  |  |  |  |  |
|  |  |  |  |  |  |  |  |  |
|  |  |  |  |  |  |  |  |  |
|  |  |  |  |  |  |  |  |  |
|  |  |  |  |  |  |  |  |  |

| **Table S9.** Associations between CTI-related indicators and the risk of CVD after exclude cancer participants. | | | | | | | | |
| --- | --- | --- | --- | --- | --- | --- | --- | --- |
|  |  |  |  |  |  |  |  |  |
|  | Model 1 | |  | Model 2 | |  | Model 3 | |
|  | HR (95% CI) | *P* value |  | HR (95% CI) | *P* value |  | HR (95% CI) | *P* value |
| **CTI-WHtR, per 1-SD increase** | 1.26 (1.20~1.33) | <0.001 |  | 1.23 (1.17~1.30) | <0.001 |  | 1.16 (1.08~1.23) | <0.001 |
| **CTI-WHtR terciles** |  |  |  |  |  |  |  |  |
| T1 | 1.0 [Ref] | |  | 1.0 [Ref] | |  | 1.0 [Ref] | |
| T2 | 1.38 (1.19~1.59) | <0.001 |  | 1.34 (1.16~1.54) | <0.001 |  | 1.29 (1.11~1.49) | 0.001 |
| T3 | 1.78 (1.55~2.04) | <0.001 |  | 1.69 (1.47~1.95) | <0.001 |  | 1.48 (1.26~1.74) | <0.001 |
| *P-*trend | 1.33 (1.25~1.42) | <0.001 |  | 1.30 (1.21~1.39) | <0.001 |  | 1.21 (1.12~1.32) | <0.001 |
| Model 1: no covariates were adjusted Model 2: Sex, Age, Smoking, Drinking were adjusted Model 3: Fully adjusted for variables, including Sex, Age, Smoking, Drinking, Marital status, Education, Residence place, Race, Liver disease, TC, HDL, UA, HbA1c, Scr, Hypertension status. Abbreviations: CVD, cardiovascular disease; BMI, body mass index; HDL-C, high-density lipoprotein cholesterol; TG, triglyceride; TC, total cholesterol; FBG, fasting blood glucose; HbA1c, hemoglobin A1c; WC, waist circumference; UA, uric acid; Scr, serum creatinine; CTI, C-reactive protein-triglycerides-glucose index; CTI-WHtR, C-reactive protein-triglycerides-glucose index - Waist-to-height ratio; CTI-BMI, C-reactive protein-triglycerides-glucose index - Body mass index; CTI-WWI, C-reactive protein-triglycerides-glucose index - Waist-to-weight index; CTI-BRI, C-reactive protein-triglycerides-glucose index - Body roundness index. | | | | | | | | |
|  |  |  |  |  |  |  |  |  |
|  |  |  |  |  |  |  |  |  |
|  |  |  |  |  |  |  |  |  |
|  |  |  |  |  |  |  |  |  |
|  |  |  |  |  |  |  |  |  |
|  |  |  |  |  |  |  |  |  |
|  |  |  |  |  |  |  |  |  |
|  |  |  |  |  |  |  |  |  |
|  |  |  |  |  |  |  |  |  |
|  |  |  |  |  |  |  |  |  |
|  |  |  |  |  |  |  |  |  |

| **Table S10.** Associations between CTI-related indicators and the risk of CVD after exclude liver disease participants. | | | | | | | | |
| --- | --- | --- | --- | --- | --- | --- | --- | --- |
|  |  |  |  |  |  |  |  |  |
|  | Model 1 | |  | Model 2 | |  | Model 3 | |
|  | HR (95% CI) | *P* value |  | HR (95% CI) | *P* value |  | HR (95% CI) | *P* value |
| **CTI-WHtR, per 1-SD increase** | 1.26 (1.20~1.33) | <0.001 |  | 1.23 (1.17~1.30) | <0.001 |  | 1.16 (1.08~1.24) | <0.001 |
| **CTI-WHtR terciles** |  |  |  |  |  |  |  |  |
| T1 | 1.0 [Ref] | |  | 1.0 [Ref] | |  | 1.0 [Ref] | |
| T2 | 1.38 (1.20~1.60) | <0.001 |  | 1.34 (1.16~1.55) | <0.001 |  | 1.29 (1.11~1.49) | 0.001 |
| T3 | 1.77 (1.54~2.03) | <0.001 |  | 1.68 (1.46~1.94) | <0.001 |  | 1.48 (1.25~1.74) | <0.001 |
| *P-*trend | 1.32 (1.24~1.42) | <0.001 |  | 1.29 (1.21~1.39) | <0.001 |  | 1.21 (1.12~1.32) | <0.001 |
| Model 1: no covariates were adjusted Model 2: Sex, Age, Smoking, Drinking were adjusted Model 3: Fully adjusted for variables, including Sex, Age, Smoking, Drinking, Marital status, Education, Residence place, Race, Cancer, TC, HDL, UA, HbA1c, Scr, Hypertension status. Abbreviations: CVD, cardiovascular disease; BMI, body mass index; HDL-C, high-density lipoprotein cholesterol; TG, triglyceride; TC, total cholesterol; FBG, fasting blood glucose; HbA1c, hemoglobin A1c; WC, waist circumference; UA, uric acid; Scr, serum creatinine; CTI, C-reactive protein-triglycerides-glucose index; CTI-WHtR, C-reactive protein-triglycerides-glucose index - Waist-to-height ratio; CTI-BMI, C-reactive protein-triglycerides-glucose index - Body mass index; CTI-WWI, C-reactive protein-triglycerides-glucose index - Waist-to-weight index; CTI-BRI, C-reactive protein-triglycerides-glucose index - Body roundness index. | | | | | | | | |
|  |  |  |  |  |  |  |  |  |
|  |  |  |  |  |  |  |  |  |
|  |  |  |  |  |  |  |  |  |
|  |  |  |  |  |  |  |  |  |
|  |  |  |  |  |  |  |  |  |
|  |  |  |  |  |  |  |  |  |
|  |  |  |  |  |  |  |  |  |
|  |  |  |  |  |  |  |  |  |
|  |  |  |  |  |  |  |  |  |
|  |  |  |  |  |  |  |  |  |
|  |  |  |  |  |  |  |  |  |
